# Deconvolution of ferromagnetic resonance spectrum of magnetic nanoparticle assembly using genetic algorithm

**DOI:** 10.1038/s41598-022-07105-7

**Published:** 2022-02-24

**Authors:** N. A. Usov, O. N. Serebryakova

**Affiliations:** 1 National University of Science and Technology «MISiS», Moscow, Russia 119049; 2 Pushkov Institute of Terrestrial Magnetism, Ionosphere and Radio Wave Propagation, Russian Academy of Sciences, IZMIRAN, Troitsk, Moscow, Russia 108480

**Keywords:** Materials science, Nanoscale materials, Magnetic properties and materials

## Abstract

The ferromagnetic resonance (FMR) spectra of dilute random assemblies of magnetite nanoparticles with cubic magnetic anisotropy and various aspect ratios are calculated using the stochastic Landau–Lifshitz equation at a finite temperature, *T* = 300 K, taking into account the thermal fluctuations of the particle magnetic moments. Particles of non-spherical shape in the first approximation are described as elongated spheroids with a given semiaxes ratio *a*/*b*, where *a* and *b* are the long and transverse semiaxes of a spheroid, respectively. A representative database of FMR spectra is created for assemblies of randomly oriented spheroidal magnetite nanoparticles with various transverse diameters *D* = 5–25 nm, moderate aspect ratios *a*/*b* = 1.0–1.8, and magnetic damping constants *κ* = 0.1, 0.2. The basic FMR spectra of assemblies with *D* = 25 nm at different aspect ratios can be considered as representatives of assemblies of single-domain magnetite nanoparticles with transverse diameters *D* > 25 nm. The database is calculated at exciting frequency *f* = 4.9 GHz (*S*-band) to clarify the details of the FMR spectrum that depend on the particle magnetic anisotropy nature. The data obtained make it possible to analyze arbitrary combined FMR spectra constructed as weighted linear combinations of FMR spectra of the base assemblies. In addition, using a genetic algorithm, the corresponding inverse problem is solved. The latter consists in determining the volume fractions of the base assemblies in some arbitrary nanoparticle assembly, which is represented by its FMR spectrum.

## Introduction

Ferromagnetic resonance (FMR) is a well-known technique^1–3^ for studying the magnetic properties of ferromagnetic materials. In some cases it allows to determine the basic magnetic characteristics of a sample under study, such as the saturation magnetization, the type of magnetic anisotropy, the directions of easy anisotropy axes and the value of magnetic anisotropy constants. This technique has been sufficiently developed for bulk single-crystals and thin magnetic films^2–4^. A significant number of theoretical and experimental studies have also been devoted to the investigation of FMR spectra of assemblies of magnetic nanoparticles^5–23^. However, it should be noted that the theory of ferromagnetic resonance is well developed for isolated single-domain magnetic nanoparticles with various types of magnetic anisotropy^1–3^. As a rule, the FMR spectrum of a single-domain nanoparticle consists of a narrow absorption line with the maximum at the resonance field *H*_*r*_, whereas the absorption line width *ΔH* is determined by the particle magnetic damping constant^1–3^. At the same time, experimental FMR spectra interpretation for magnetic nanoparticles assemblies causes a difficulty^21^. This is due to the wide distributions of the nanoparticle sizes and shapes in a real assembly. Namely, due to the influence of shape anisotropy, the particles of the same chemical composition are usually characterized by a combined type of magnetic anisotropy^12,15,19,24^. In addition, the spatial orientation of nanoparticles in the assembly is random, as a rule. This leads to a further expansion of the resonance fields *H*_*r*_ of individual nanoparticles. Moreover, the FMR spectra of sufficiently small nanoparticles are greatly influenced by thermal fluctuations of the particle magnetic moments^7,8,19,20^. Finally, strong magneto-dipole (MD) interaction in a dense nanoparticle assembly affects the dynamics of the particle magnetic moments^14,18,22,23^. Under the influence of all these factors, the experimental FMR spectrum of an assembly of magnetic nanoparticles turns out to be very smoothed and broad^18,19,21,22^.

At present the evolution of the FMR spectra of individual non-interacting nanoparticles, depending on changes in their sizes and anisotropy constants, the temperature of the assembly, and other factors has been well studied theoretically^5–11,15,17,20^. At the same time, the problem of determining the magnetic and geometric characteristics of nanoparticles from experimental FMR spectra has not yet received a satisfactory solution. This diminishes the importance of the FMR technique when applied to the study of the properties of magnetic nanoparticle assembly.

This paper is devoted to the calculation and interpretation of FMR spectra of dilute assemblies of magnetite nanoparticles, which are of interest for research in biomedicine^25,26^ and paleomagnetism^27–29^, since magnetite is part of the fossil remains of living organisms. The calculation of the FMR spectra of random assemblies of magnetite nanoparticles is carried out by solving the stochastic Landau–Lifshitz equation^30–33^ at a finite temperature, *T* = 300 K, taking into account the thermal fluctuations of the particle magnetic moments. Similar approach for random assembly of single-domain nanoparticles with uniaxial anisotropy was developed previously in Refs.^13,17^. The effect of the magneto- dipole interaction on the FMR spectrum of spherical magnetite nanoparticles is also investigated for an assembly of dilute nanoparticle clusters with different filling densities. The problem of decoding the FMR spectrum of a random assembly of magnetite nanoparticles is solved in two steps. First, using numerical simulation, a representative database of FMR spectra is created for assemblies of magnetite nanoparticles with various transverse diameters, aspect ratios and magnetic damping constants. The data obtained make it possible to trace in detail the change in the shape of the FMR spectrum of the assembly with a change in each of the parameters indicated. This also makes it possible to analyze arbitrary combined FMR spectra obtained as weighted linear combinations of FMR spectra of the base assemblies. At the second stage, the corresponding inverse problem is solved using a genetic algorithm^34–36^. The latter consists in determining the volume fractions of the base assemblies in some arbitrary assembly, which is represented by its FMR spectrum.

## Results and discussion

The calculation of the FMR spectra of assemblies of magnetite nanoparticles are carried out under the assumption that the particles are single-crystal, have a saturation magnetization *M*_*s*_ = 450 emu/cm^3^, and are characterized by a cubic type of magneto-crystalline anisotropy with an anisotropy constant *K*_*c*_ = − 10^5^ erg/cm^3^^37^. In addition, we take into account the influence of the shape anisotropy energy that appears due to the deviation of the particle shape from that of an ideal sphere. Particles of non-spherical shape in the first approximation are described as elongated spheroids with a given semiaxes ratio *a*/*b*, where *a* and *b* are the long and transverse semiaxes of a spheroid, respectively. The uniaxial shape anisotropy energy increases as a function of *a*/*b* ratio and is added to the initial cubic anisotropy energy. It is reasonable to assume that in a real assembly the direction of the symmetry axis of the spheroid with respect to the axes of the cubic magnetic anisotropy is arbitrary. Besides, for a randomly oriented assembly, the orientations of the long axes of the spheroids with respect to the direction of the external magnetizing field *H*_0_ is also random. The latter, for definiteness, is assumed to be directed along the Z-axis of the Cartesian coordinates. Then, without loss of generality one can assume that a weak alternating (ac) magnetic field of frequency *f* that excites resonance is applied along the *X* axis.

It is clear that a FMR spectrum of any dilute assembly of nanoparticles can be obtained as a linear superposition of a sufficiently complete set of basic FMR spectra. The basic FMR spectra in this work are FMR spectra of assemblies of randomly oriented spheroidal magnetite nanoparticles with a given value of the transverse diameter *D* = 2*b*, aspect ratio *a*/*b*, and magnetic damping constant *κ*. Our calculations show that at a given temperature *T* = 300 K, the indicated parameters (*D*, *a*/*b*, *κ*) completely determine the shape of the FMR spectrum of the basic magnetite nanoparticle assembly.

### Basic FMR spectra

In the calculated database of FMR spectra the transverse diameters of particles vary in the range of 5–25 nm, the semiaxes ratio of the spheroids changes from *a*/*b* = 1.0 to *a*/*b* = 1.8, the magnetic damping constant takes the values *κ* = 0.1, 0.2. The frequency of the ac magnetic field exciting the resonance is chosen to be *f* = 4.9 GHz (*S*-band), since at this excitation frequency the ferromagnetic resonance is observed at lower values of the external field *H*_0_. As a result, the details of the FMR spectrum that depend on particle magnetic anisotropy manifest themselves more clearly.

**Figure 1:**
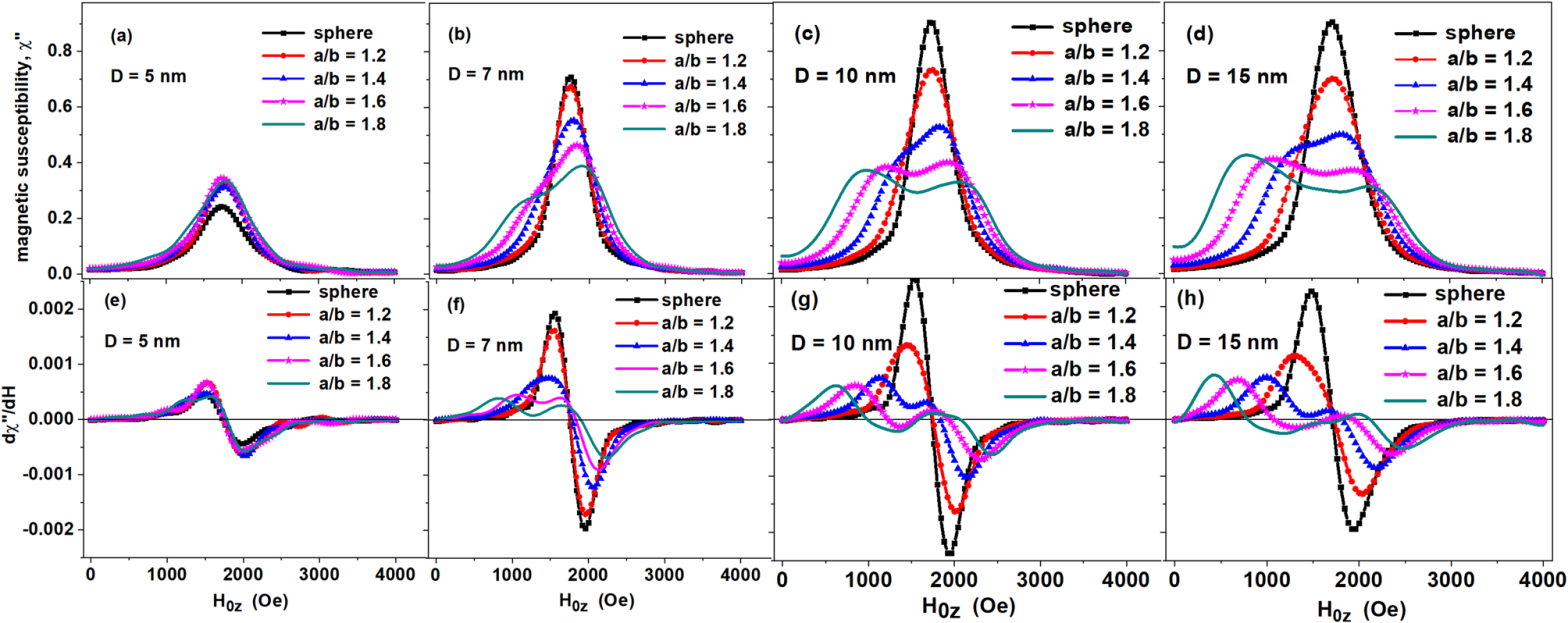
Dependence of the FMR spectrum of a dilute randomly oriented assembly of magnetite nanoparticles on the aspect ratio *a*/*b* = 1.0–1.8 for different transverse particle diameters: (**a**, **e**) *D* = 5 nm; (**b**, **f**) *D* = 7 nm; (**c**, **g**) *D* = 10 nm and (**d**, **h**) *D* = 15 nm. FMR excitation frequency *f* = 4.9 GHz, magnetic damping constant *κ* = 0.1, temperature *T* = 300 K.

Figure 1 shows the change in the basic FMR spectra of dilute randomly oriented assemblies of magnetite nanoparticles depending on the aspect ratio *a*/*b* for the transverse diameters of particles in the range *D* = 5–15 nm. The top panels in Fig. 1 show the magnetic susceptibility of the assemblies as a function of the applied external field *H*_0_, the lower panels show the derivative of the magnetic susceptibility with respect to the magnetic field. Further, the shape of the FMR spectrum will mean precisely the dependence of the magnetic susceptibility of the assembly on the applied external magnetic field, which is the fundamental high-frequency characteristic of the magnetic nanoparticle assembly.

As Fig. 1 shows, the effect of thermal fluctuations has the strongest influence on the FMR spectra of nanoparticles with small transverse diameters, *D* ≤ 7 nm, while with an increase in the transverse diameter from 10 to 15 nm the change in the FMR spectra is relatively small. As one can see from the upper panels in Fig. 1, for nanoparticles with a transverse diameter *D* ≥ 7 nm the FMR spectrum of the assembly becomes double humped with an increase in the *a*/*b* ratio, which is associated with an increase in the particle shape anisotropy energy. In addition, the FMR spectrum width also increases as a function of *a*/*b* ratio.

The shapes of the spectrum maxima change with an increase in the particle diameter in the range *D* = 7–15 nm, which is associated with a decrease in the effect of thermal fluctuations. However, for particles with diameters *D* ≤ 5 nm the dependence of the FMR spectrum on the aspect ratio becomes weak. For small particles the FMR spectrum has a single maximum in the entire range of aspect ratios *a*/*b* studied.

The data presented in Fig. 1 show that in a general case the FMR spectrum of magnetite nanoparticle assembly cannot be characterized by a single maximum with a fixed resonance field *H*_*r*_. Such a description is qualitatively valid only for nanoparticles whose shape does not differ much from spherical, *a*/*b* ≤ 1.3. It is interesting to note that the behavior of FMR spectrum of non oriented assembly of magnetite nanoparticles as a function of particle aspect ratio resemble that of random assembly of uniaxial nanoparticles^17^ depending on the value of uniaxial anisotropy constant. This is because the uniaxial shape anisotropy energy dominates in the total anisotropy energy of magnetite nanoparticles with aspect ratio *a*/*b* ≥ 1.5.

**Figure 2:**
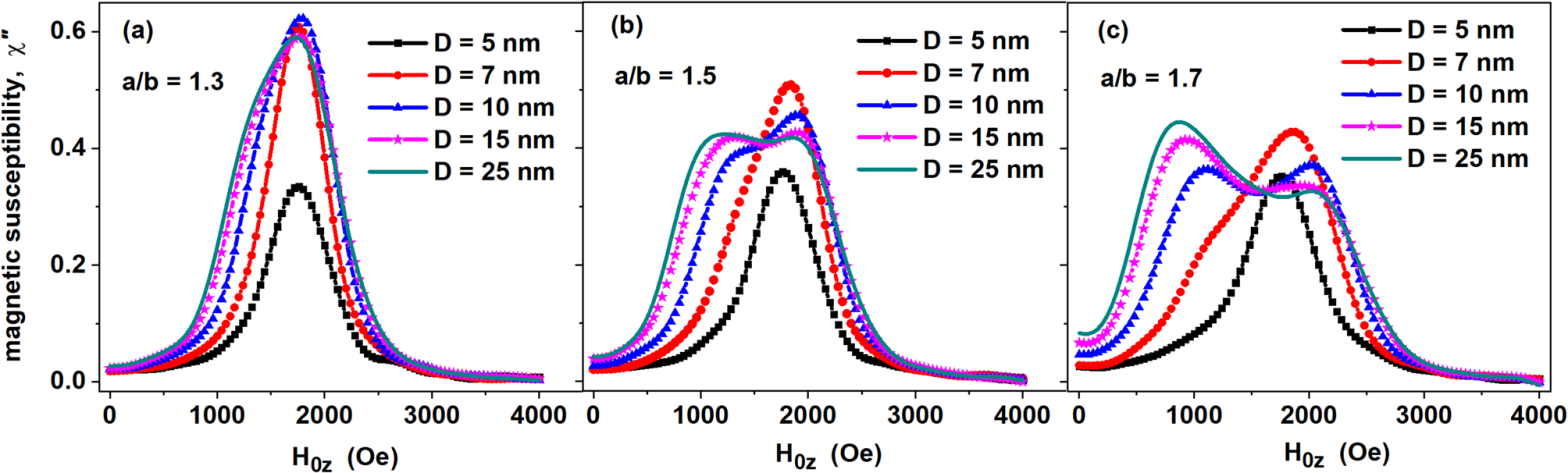
Dependence of the FMR spectrum of a dilute randomly oriented assembly of magnetite nanoparticles on the transverse particle diameter *D* = 2*b* for various aspect ratios: (**a**) *a*/*b* = 1.3; (**b**) *a*/*b* = 1.5; (**c**) *a*/*b* = 1.7. FMR excitation frequency *f* = 4.9 GHz, magnetic damping constant *κ* = 0.1, *T* = 300 K.

Figure 2 shows the change in the shape of the FMR spectrum as a function of the transverse diameter for a fixed aspect ratio of the particles. Note that the FMR spectra of particles with diameters *D* = 15 and 25 nm in all the panels shown practically coincide. Calculations show that with a further increase in the transverse particle diameter, *D* > 25 nm, the FMR spectrum of the assembly with the given *a*/*b* and *κ* values does not change. Thus, the basic FMR spectra of particles with *D* = 25 nm at different aspect ratios *a*/*b* = 1.0–1.8 can be considered as representatives of all single-domain magnetite nanoparticle assemblies with transverse diameters *D* > 25 nm. Significant changes in the spectra, when the double humped nature of the spectrum changes to a single maximum, occur in the range of diameters *D* < 10 nm.

**Figure 3:**
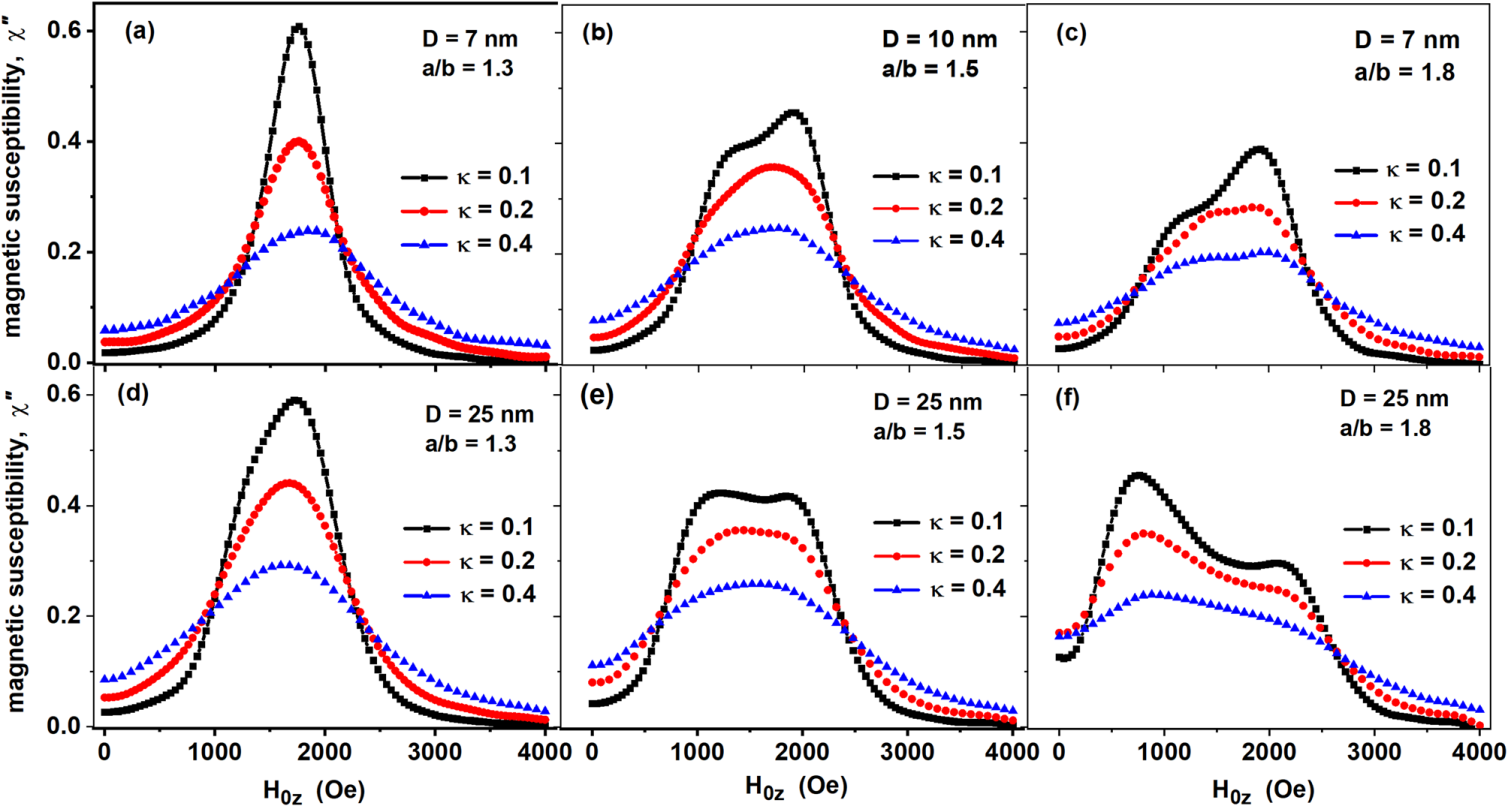
Dependence of the FMR spectrum on the magnetic damping constant *κ* = 0.1, 0.2, and 0.4 for different transverse diameters *D* and aspect ratios of particles *a*/*b*. FMR excitation frequency *f* = 4.9 GHz, *T* = 300 K.

The bottom panels in Fig. 3 show the evolution of the FMR spectra of magnetite nanoparticles of sufficiently large transverse diameters, *D* ≥ 25 nm, with an increase in the magnetic damping constant *κ* for particles with aspect ratios *a*/*b* = 1.3, 1.5, and 1.8, respectively. It is obvious that an increase in the magnetic damping constant significantly decreases the amplitude of the FMR signal and smoothes the spectrum, transforming it from double-belled to single-belled for the aspect ratios of particles *a*/*b* ≥ 1.5. However, an increase in *κ* only weakly affects the FMR spectrum width. The top panels in Fig. 3 show the effect of thermal fluctuations on the shape of the FMR spectrum for assemblies of nanoparticles with small transverse diameters, *D* ≤ 10 nm. Comparing the shape of the spectra in panels (a) and (d) in Fig. 3 one can see that for particles with moderate aspect ratios, *a*/*b* = 1.3, the spectral width at half maximum decreases under the influence of thermal fluctuations. At the same time, for particles with aspect ratios *a*/*b* ≥ 1.5, the shape of the FMR spectrum changes significantly under the influence of thermal fluctuations, which is especially noticeable for assemblies with *κ* = 0.1.

**Figure 4:**
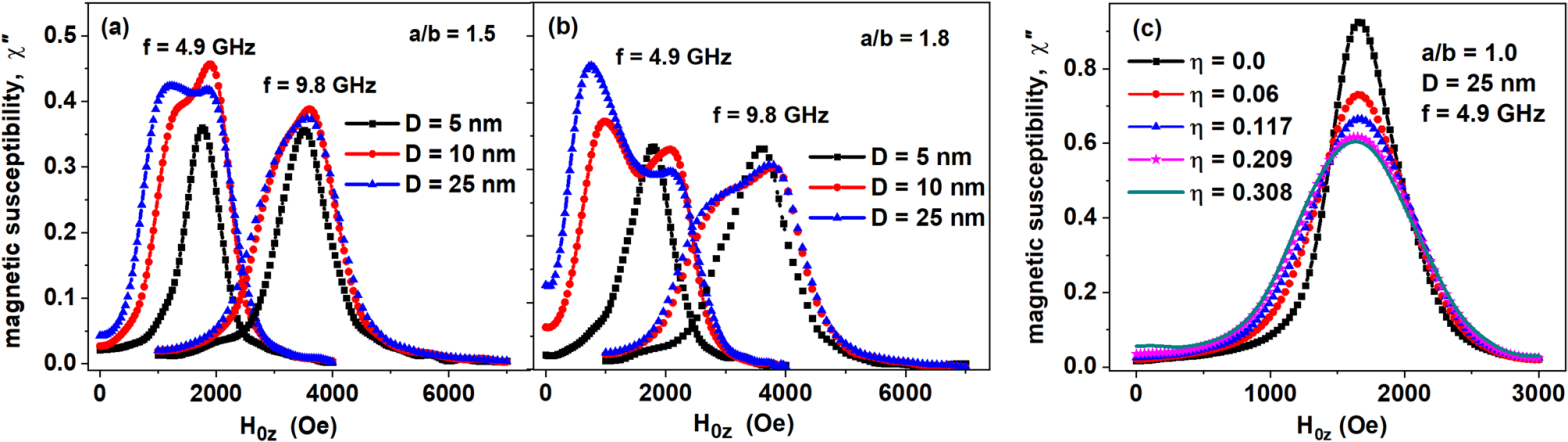
(**a**, **b**) Change in the FMR spectrum with increase of the excitation frequency from *f* = 4.9 GHz to 9.8 GHz. (**c**) influence of magnetic dipole interaction on the FMR spectrum in dense clusters of spherical magnetite nanoparticles. The magnetic damping constant *κ* = 0.1, *T* = 300 K.

It was mentioned above that the correct choice of the excitation frequency is important to reveal the characteristic details of the FMR spectrum. Actually, Fig. 4a and b show the change in the FMR spectrum in assemblies of magnetite nanoparticles with aspect ratios *a*/*b* = 1.5 and 1.8, respectively, with an increase in the excitation frequency from *f* = 4.9 GHz to 9.8 GHz (*X*-band).

Obviously, with an increase in the excitation frequency, the domain of observation of ferromagnetic resonance shifts to the range of high magnetizing fields. As a result, some characteristic details of the FMR spectrum are lost, since in a large magnetizing field the Zeeman energy is dominant in comparison with the magnetic anisotropy energy of the nanoparticles. Indeed, as Fig. 4a,b show, at the excitation frequency *f* = 4.9 GHz, one can clearly see the transformation of a single-belled FMR spectrum into a double-belled one with an increase in the transverse diameter of particles from 5 to 25 nm, while at the frequency *f* = 9.8 GHz this tendency is less noticeable.

For completeness of the study we also investigated the change in the FMR spectrum of spherical magnetite nanoparticles under the action of the magneto- dipole (MD) interaction depending on the filling density of nanoparticle clusters. The cluster filling density is characterized^38^ by the ratio *η* = *N*_*p*_*V*/*V*_*cl*_, where *V* is the volume of a particle and *V*_*cl*_ is the volume of a quasi-spherical cluster containing *N*_*p*_ nanoparticles. Calculations of FMR spectra were performed for dilute assemblies of clusters containing *N*_*p*_ = 60 of spherical magnetite nanoparticles. As Fig. 4c shows, with an increase in the cluster filling density in the range *η* = 0.06–0.308, the maximum of the FMR spectrum noticeably decreases and shifts to lower magnetizing fields, while the width of the spectrum increases. Consequently, the effect of strong MD interaction in dense assemblies of magnetic nanoparticles leads to additional deformation of the FMR spectrum. This effect is worth to be studied separately.

### Genetic algorithm

The Supplemented Material presents a set of basic FMR spectra of assemblies of magnetite nanoparticles in the form of dependences of the magnetic susceptibility of the assemblies $\chi^{\prime\prime}\left( {D,a/b,H_{0z} } \right)$ on the external magnetic field *H*_0*z*_ at an excitation frequency *f* = 4.9 GHz and at a temperature of *T* = 300 K. The basic FMR spectra of dilute randomly oriented assemblies of nanoparticles are calculated for magnetic damping constants *κ* = 0.1 and 0.2 and are presented in Supplementary Tables 1 and 2, respectively. Derivatives of FMR spectra with respect to the applied magnetic field, $d\chi^{\prime\prime}/dH$, can be obtained from these Supplementary Tables by numerical differentiation. In the calculations performed, the transverse diameter of magnetite particles takes the values *D* = 5, 7, 10, 15, and 25 nm, the aspect ratios vary with a step of 0.1 from *a*/*b* = 1.0 (sphere) to *a*/*b* = 1.8 (moderately elongated particles). As noted earlier, at a fixed temperature *T* = 300 K the FMR spectra of particles with *D* = 25 nm can also be considered as representatives of the spectra of single-domain assemblies of nanoparticles with transverse diameters *D* > 25 nm, since at sufficiently large transverse sizes the effect of thermal fluctuations on the FMR spectrum is negligible.

The first column of Supplementary Tables 1 and 2 gives the values of the external magnetic field, which in these calculations varied with a step of 40 Oe. These values can be written as *H*_0*z*_ = 40(*j* − 1), where the integer *j* runs from 1 to 101. The subsequent columns of Supplementary Tables 1 and 2 show the corresponding values of the magnetic susceptibility $\chi^{\prime\prime}$, that are written in the following order: *a*/*b* = 1.0, *D* = 5, 7, 10, 15 and 25 nm, then *a*/*b* = 1.1, *D* = 5, 7, 10, 15 and 25 nm, etc. It is convenient to enumerate the columns of the Supplementary Tables 1 and 2, starting from the second one, with the index *i*, which runs through the values *i* = 1–45. Thus, the columns with numbers *i* = 1–5 correspond to the values *a*/*b* = 1.0, and the transverse diameters *D* = 5, 7, 10, 15, and 25 nm, respectively, columns *i* = 6–10 correspond to the values *a*/*b* = 1.1, and diameters *D* = 5, 7, 10, 15 and 25 nm, etc. As a result, the values of the magnetic susceptibility written in the form of Supplementary Tables 1 and 2 represent matrixes $\chi^{\prime\prime}\left( {i,j} \right)$.

Obviously, the FMR spectrum of a composite dilute assembly of magnetite nanoparticles can be obtained as a weighted linear combination of the basic FMR spectra of the form

1 $$ \overline{\chi^{\prime\prime}}\left( j \right) = \sum\limits_{i = 1}^{45} {\lambda_{i} \chi^{\prime\prime}\left( {i,j} \right)} $$

where the coefficients *λ*_*i*_, the sum of which is equal to 1, are the volume fractions of magnetite nanoparticles with the corresponding values (*D*, *a*/*b*)_*i*_ in this composite assembly.

**Figure 5:**
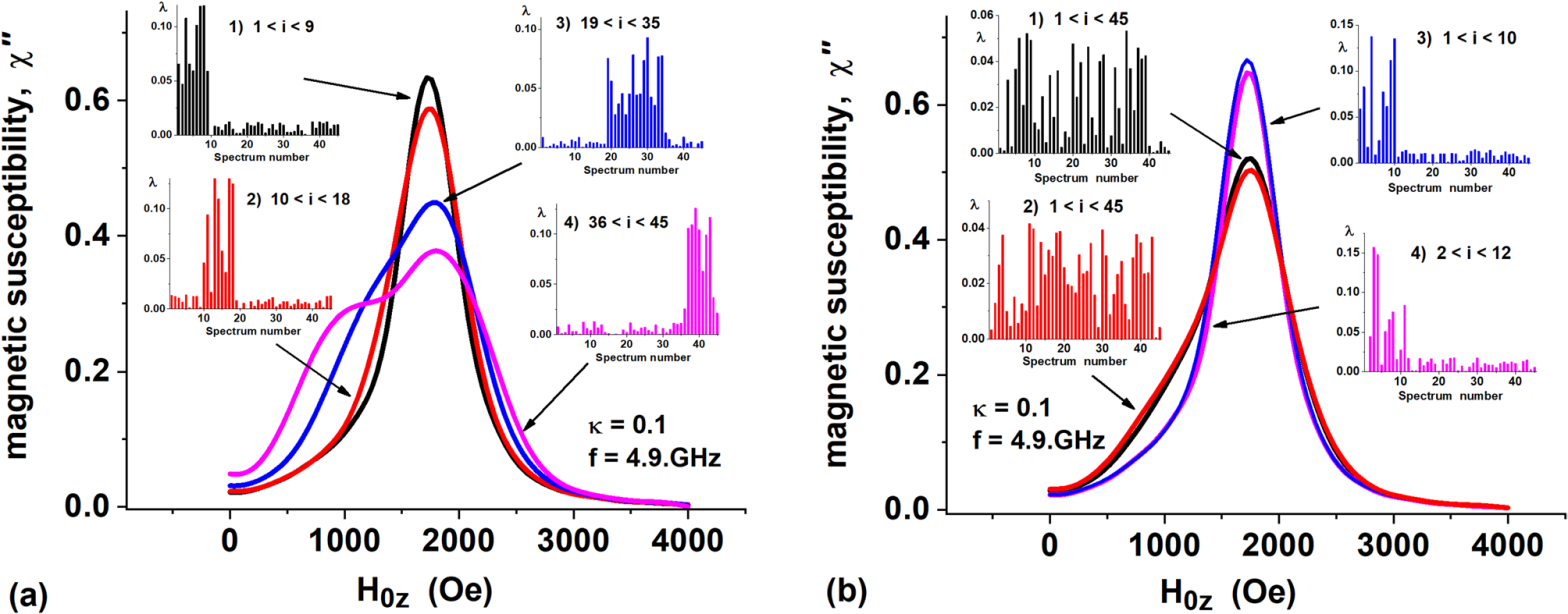
Composite FMR spectra constructed on the basis of basic FMR spectra presented in Supplementary Table 1. (**a**) evolution of the FMR spectrum of composite assemblies having the distribution of volume fractions *λ*_*i*_ shown in the insets (1)–(4); (**b**) comparison of composite FMR spectra for assemblies with uniformly distributed volume fractions, cases (1), (2) and assemblies consisting mainly of quasi-spherical nanoparticles, cases (3), (4).

First of all, it is interesting to find out how the FMR spectrum of a composite assembly changes depending on the distribution of its constituent fractions *λ*_*i*_, *i* = 1, 2,… 45. The corresponding illustrative calculations are presented in Fig. 5.

Figure 5a shows FMR spectra of composite assemblies constructed from different random sets of particle volume fractions *λ*_*i*_, shown in insets (1)–(4). The heights of the bars in the insets show the values of the corresponding coefficients *λ*_*i*_ in the given linear combination. For insert (1) the maximum values of the coefficients *λ*_*i*_ are approximately uniformly distributed in the interval 1 ≤ *i* ≤ 9. Outside this interval the coefficients *λ*_*i*_ are also random and close to zero. Similarly, for insert (2) the maximum values of *λ*_*i*_ are concentrated in the interval 10 ≤ *i* ≤ 18, for insert (3) in the interval 19 ≤ *i* ≤ 36, and for insert (4) in the interval 36 ≤ *i* ≤ 45, respectively.

It is easy to see that in the first case, 1 ≤ *i* ≤ 9, the composite assembly contains mainly quasi-spherical particles with aspect ratios *a*/*b* = 1.0–1.1, in the second case, the particle aspect ratios correspond to the interval *a*/*b* = 1.2–1.3, etc. As Fig. 5a shows the FMR spectrum of the composite assembly changes noticeably depending on the volume fraction distribution which corresponds to different intervals of *a*/*b* values.

At the same time, as Fig. 5b shows, the distribution of the volume fractions within a given interval of *a*/*b* values does not significantly affect the shape of the FMR spectrum of a composite assembly. Indeed, in Fig. 5b the insets (1) and (2) show different random distributions of the coefficients *λ*_*i*_, homogeneous over the entire range of values 1 ≤ *i* ≤ 45. One can see that these two different distributions of volume fractions correspond to very similar FMR spectra. Similarly, insets (3) and (4) show two different distributions of volume fractions in assemblies of quasi-spherical nanoparticles, for which the coefficients *λ*_*i*_ are concentrated in the intervals 1 ≤ *i* ≤ 10 and 2 ≤ *i* ≤ 12, respectively. Again, despite the difference in the distribution of volume fractions for cases (3) and (4), the corresponding FMR spectra presented in Fig. 5b turn out to be very close.

The previous consideration shows examples of the FMR spectra of dilute composite assemblies obtained using known volume fractions of its constituent basic assemblies. It is clear that if a method for solving the so-called inverse problem were developed, the FMR technique would be much more useful for applications. The inverse problem consists in determining the volume fractions *λ*_*i*_ from the known FMR spectrum of a dilute composite assembly.

In this work this problem is solved by using the simplest version of the genetic algorithm^34,35^, which is described in the Methods section. Despite the aforementioned weak sensitivity of the FMR spectrum of the composite assembly to the distribution of the coefficients *λ*_*i*_ in the given intervals of *a*/*b* values, the used version of the genetic algorithm demonstrates the ability, in a relatively small number of iterations, to reproduce the distribution of volume fractions *λ*_*i*_, which is very close to some arbitrary given distribution *λ*_*i*_^(0)^.

**Figure 6:**
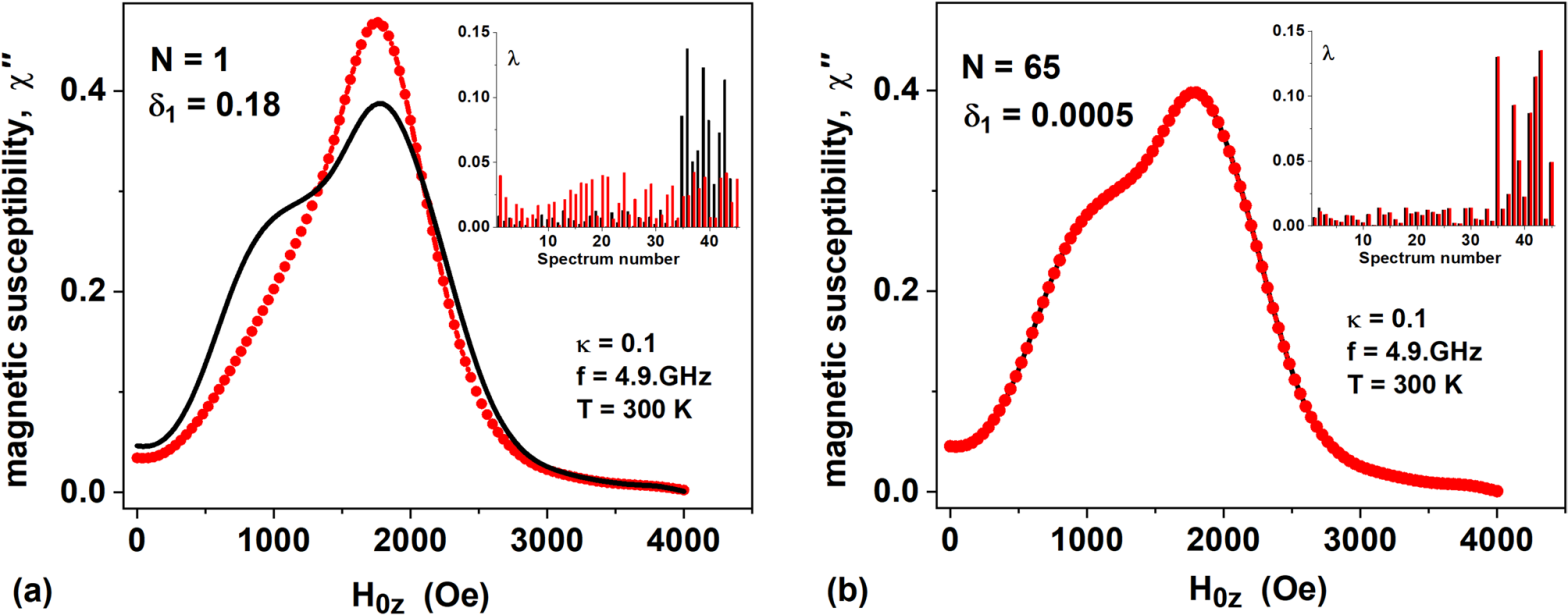
Application of a genetic algorithm to determine the volume fractions of basic assemblies of magnetite nanoparticles from a given FMR spectrum of a certain dilute composite assembly: (**a**) the first iteration of the genetic algorithm, *N* = 1, (**b**) the final iteration, *N* = 65. The insets in the figures show the given distribution of volume fractions *λ*_*i*_^(0)^ (black bars) and the current distribution of volume fractions *λ*_*i*_ (red bars), obtained at the first and final iterations of the genetic algorithm, respectively.

In Fig. 6a the solid black curve shows the FMR spectrum of a certain dilute composite assembly of magnetite nanoparticles, and the black bars in the inset in this figure show the specified distribution of volume fractions *λ*_*i*_^(0)^ for this spectrum. The red bars in the inset in Fig. 6a show the initial distribution of volume fractions *λ*_*i*_ (*N* = 1), generated by the genetic algorithm at the first iteration. The corresponding initial FMR spectrum is shown in Fig. 6a with red dots. As Fig. 6a shows, the initial distribution of volume fractions and the initial shape of the FMR spectrum, formed at the first iteration by the genetic algorithm, are very far from the specified distribution of volume fractions *λ*_*i*_^(0)^. Accordingly, the residual of the genetic algorithm, which estimates the closeness of the current and the desired distributions, at the initial stage is very large, *δ*_1_ *N* = 1) = 0.18. However, in the course of iterations, the current distribution of volume fractions quickly approaches the desired one. Figure 6b shows that already at iteration *N* = 65, when the algorithm residual decreases to the value *δ*_1_ (*N* = 65) = 0.0005, the distribution of volume fractions *λ*_*i*_ (*N* = 65) turns out to be very close to the given distribution *λ*_*i*_^(0)^. Moreover, calculations show that the FMR spectrum formed by the genetic algorithm is practically indistinguishable from the specified FMR spectrum even at the algorithm residual values *δ*_1_ < 0.01.

Similar results were also obtained when the genetic algorithm was applied to the set of FMR spectra presented in Supplementary Table 2. It should be noted that the number of iterations *N*_*max*_ required for the genetic algorithm to obtain the specified FMR spectrum of the composite assembly with the required accuracy depends significantly on the initial distribution that was randomly generated at its initial stage. In some cases, the variation in *N*_*max*_ can be several hundred. In addition, it is well known^35^ that in the case of an unsuccessfully formed initial state, the convergence of the algorithm may turn out to be very slow, or even absent. In this case it is recommended to stop the current calculations and to create a new initial state. As a rule, for new initial state the rate of evolution of the volume fraction distribution to a given distribution is acceptable. Anyway, it is found that if the genetic algorithm converges, it always leads exactly to the given distribution of volume fractions *λ*_*i*_^(0)^.

## Conclusions

Magnetic nanoparticle assembly is known to be a complex physical system. Its properties are determined by both geometric and magnetic particle parameters. The standard measurement of a quasi-static hysteresis loop of an assembly makes it possible to determine the saturation magnetization of the particles, the coercive force, and the initial magnetic susceptibility of the assembly. However, many of the important microscopic characteristics of the assembly, that is, the particle size and shape distribution, the type of magnetic anisotropy of the particles, the distribution of the directions of the easy anisotropy axes in space, remain unknown. So do the dynamic characteristics of the particles, such as the magnetic damping constant and the characteristic particle relaxation time. The geometric characteristics of the particles are usually determined by rather expensive electron microscopic methods^21,22^. In turn, FMR spectra measurement opens the way to assessing the dynamic characteristics of nanoparticles. However, the FMR technique as applied to assemblies of nanoparticles would be in greater demand if an effective method for a detailed analysis of complex FMR spectra of magnetic nanoparticle assemblies were developed and validated.

In this work it is proposed to use the genetic algorithm^34–36^ for the analysis of FMR spectra of dilute assemblies of magnetite nanoparticles, which are widely used in biomedicine^24,25^ and are interesting for paleomagnetic studies^27–29^. The genetic algorithm is known as a very productive way to iterate over a large number of feasible options when searching for an option that meets certain criteria. To implement this technique in the paper, we calculated a representative set of basic FMR spectra of dilute magnetite nanoparticle assemblies with fixed values of the transverse diameter *D* = 5–25 nm, the aspect ratio *a*/*b* = 1.0–1.8, and the magnetic damping constants *κ* = 0.1, 0.2. Using the constructed database, the FMR spectrum of a certain composite dilute assembly of magnetite nanoparticles can be represented with high accuracy as a linear combination of basic FMR spectra. The coefficients determined by the genetic algorithm in the linear combination constructed are the volume fractions of the basic assemblies in the original composite assembly of magnetite nanoparticles.

Due to limited scope of the article, the results of applying the genetic algorithm are demonstrated only for the case *κ* = 0.1. This value was chosen because, according to some experimental data, it seems to be the most plausible for iron oxide nanoparticle assemblies. Nevertheless, similar results for application of genetic algorithm were also obtained for *κ* = 0.2. At the final stage of this study it is supposed to have basic FMR spectra of magnetite nanoparticles for a sufficiently representative set the magnetic damping constants. Thus, a researcher gets the opportunity, on the basis of the measured FMR spectrum of a dilute assembly, to estimate the size and shape distribution of the assembly particles, and, in addition, to estimate the most appropriate value of the magnetic damping constant.

Unfortunately, this technique is hardly applicable to the analysis of FMR spectra of dense assemblies of magnetic nanoparticles, since the strong MD interaction of particles in dense assemblies has a noticeable additional effect on the shape of the FMR spectrum. On the other hand, calculations show that the genetic algorithm can be successfully used to analyze the FMR spectra of dilute assemblies of nanoparticles of other types, for example, nanoparticles with uniaxial magnetic anisotropy.

## Methods

### Calculation of basic FMR spectra

Let us consider a dilute assembly of *N*_*p*_ spheroidal magnetite nanoparticles with given geometric parameters *D*, *a*/*b*, and a fixed value of the magnetic damping constant *κ*. In a randomly oriented assembly, the magneto-crystalline anisotropy energy of magnetite nanoparticles is

2 $$ W_{mc} = K_{c} V\sum\limits_{i = 1}^{{N_{p} }} {\left( {\left( {\vec{\alpha }_{i} \vec{e}_{1i} } \right)^{2} \left( {\vec{\alpha }_{i} \vec{e}_{2i} } \right)^{2} + \left( {\vec{\alpha }_{i} \vec{e}_{1i} } \right)^{2} \left( {\vec{\alpha }_{i} \vec{e}_{3i} } \right)^{2} + \left( {\vec{\alpha }_{i} \vec{e}_{2i} } \right)^{2} \left( {\vec{\alpha }_{i} \vec{e}_{3i} } \right)^{2} } \right)} $$

where *V* = 4*πab*^2^/3 is the volume of spheroidal particle, ***α***_*i*_ is the unit magnetization vector and (***e***_1*i*_, ***e***_2*i*_, ***e***_3*i*_) is the set of orthogonal unit vectors that determine the spatial orientations of the cubic easy anisotropy axes of *i*-th nanoparticle of the assembly. Since the axes of symmetry of spheroidal nanoparticles are assumed to be randomly and uniformly distributed in space, the shape anisotropy energy of the assembly can be written as

3 $$ W_{Sh} = K_{Sh} V\sum\limits_{i = 1}^{{N_{p} }} {\left( {1 - \left( {\vec{\alpha }_{i} \vec{n}_{i} } \right)^{2} } \right)} $$

where *K*_*Sh*_ is the shape anisotropy constant and ***n***_*i*_ is the unit vector along the direction of elongation of *i*-th nanoparticle. The shape anisotropy constant is given by^39^

4 $$ K_{Sh} = M_{s}^{2} \left( {\pi - {{3N_{a} } \mathord{\left/ {\vphantom {{3N_{a} } 4}} \right. \kern-\nulldelimiterspace} 4}} \right);\;\;\;\;\;N_{a} = 2\pi \frac{{1 - \varepsilon^{2} }}{{\varepsilon^{3} }}\left( {\ln \frac{1 + \varepsilon }{{1 - \varepsilon }} - 2\varepsilon } \right);\;\;\;\;\varepsilon = \sqrt {1 - \left( {{b \mathord{\left/ {\vphantom {b a}} \right. \kern-\nulldelimiterspace} a}} \right)^{2} } $$

Here *N*_*a*_ is the demagnetizing factor along the long nanoparticle axis.

Zeeman energy of the assembly in a constant magnetic field ***H***_0_ = (0,0,*H*_0*z*_), and a weak ac magnetic field with an amplitude ***H***_1_ = (*H*_1*x*_,0,0) is given by

5 $$ W_{Z} = - M_{s} V\sum\limits_{i = 1}^{{N_{p} }} {\left( {\alpha_{ix} H_{1x} \sin \left( {\omega t} \right) + \alpha_{iz} H_{0z} } \right)} $$

where *ω* = 2*πf* is the angular frequency of the ac magnetic field.

Dynamics of the unit magnetization vector $\vec{\alpha }_{i}$ of *i*-th single-domain nanoparticle of the assembly is governed by stochastic Landau–Lifshitz equation^30–33^

6 $$ \frac{{\partial \vec{\alpha }_{i} }}{\partial t} = - \gamma_{1} \vec{\alpha }_{i} \times \left( {\vec{H}_{ef,i} + \vec{H}_{th,i} } \right) - \kappa \gamma_{1} \vec{\alpha }_{i} \times \left( {\vec{\alpha }_{i} \times \left( {\vec{H}_{ef,i} + \vec{H}_{th,i} } \right)} \right), \;\;\;\;\;\;i = \, 1,2, \ldots N_{p} , $$

where *γ* is the gyromagnetic ratio, *γ*_1_ = *γ*/(1 + *κ*^2^), $\vec{H}_{ef,i}$ is the effective magnetic field and $\vec{H}_{th,i}$ is the thermal field. The effective magnetic field acting on a separate nanoparticle can be calculated as a derivative of the total energy *W* = *W*_*mc*_ + *W*_*Sh*_ + *W*_*Z*_

7 $$ \vec{H}_{ef,i} = - \frac{\partial W}{{M_{s} V\partial \vec{\alpha }_{i} }} $$

The thermal fields $\vec{H}_{th,i}$ acting on various nanoparticles of the assembly are statistically independent, with the following statistical properties^30^ of their components.

8 $$ \left\langle {H_{th.i}^{(\alpha )} \left( t \right)} \right\rangle = 0;\;\;\;\;\;\left\langle {H_{th,i}^{(\alpha )} \left( t \right)H_{th,i}^{(\beta )} \left( {t_{1} } \right)} \right\rangle = \frac{{2k_{B} T\kappa }}{{\gamma M_{s} V}}\delta_{\alpha \beta } \delta \left( {t - t_{1} } \right),\;\;\;\;\,\alpha ,\beta = \left( {x,y,z} \right). $$

Here *k*_*B*_ is the Boltzmann constant, *δ*_*αβ*_ is the Kroneker symbol, and *δ*(*t*) is the delta function.

It is well known^26,38^ that the power absorbed by the assembly per unit time and per unit volume is proportional to the area of the assembly hysteresis loop

9 $$ P = M_{s} f\oint \frac{1}{N_{p}}{\sum\limits_{i = 1}^{{N_{p} }} {\vec{\alpha }_{i} } d\vec{H}} = M_{s} f\oint {\vec{m}d\vec{H}} .$$

where ***m*** is the reduced magnetic moment of the assembly. To numerically calculate the power absorbed by an assembly of superparamagnetic nanoparticles in ac magnetic field ***H***_1_(*t*), it is convenient to rewrite Eq. (9) in the form of the time-averaged integral

10 $$ P = M_{s} \frac{1}{\Delta t}\int\limits_{t}^{t + \Delta t} {m_{x} } \frac{{dH_{1x} }}{dt}dt $$

where Δ*t* is a certain time interval significantly exceeding the period of oscillations of the ac magnetic field, *τ* = 2π/ω. On the other hand, using the small amplitude of the ac magnetic field, the same quantity can be expressed in terms of the imaginary part of the magnetic susceptibility of the assembly^3,40^

11 $$ P = \pi f\chi^{\prime\prime}\left( {H_{0z} ,f} \right)H_{1x}^{2} $$

Comparison of Eqs. (10) and (11) makes it possible to obtain the imaginary part of the magnetic susceptibility $\chi^{\prime\prime}\left( {H_{0z} ,f} \right)$ of the assembly as a function of the magnetizing field component *H*_0*z*_.

The calculation of the absorbed power according to Eq. (10) was carried out in this work for randomly oriented basic assemblies of magnetite nanoparticles with fixed parameters *D*, *a*/*b*, and *κ*. The calculation results were averaged over a fairly large number of independent experiments, *N*_*exp*_ = 100. In every experiment a new assembly of *N*_*p*_ = 100 non interacting spheroidal magnetite nanoparticles with random directions of the cubic anisotropy axes and random directions of the long axes of the spheroids was created. The frequency of the ac magnetic field exciting the resonance was *f* = 4.9 GHz, or *f* = 9.8 GHz, the amplitude of the ac magnetic field was *H*_1*x*_ = 10 Oe. The numerical time step was 1/30 of the characteristic precession period of the unit magnetization vectors. The full time interval of calculations covered at least 200 periods of the ac magnetic field, whereas time averaging of the integral in Eq. (10) occurred over the last quarter of the total number of periods, when the dynamics of unit magnetization vectors became stationary. Thus, the time interval Δ*t* in Eq. (10) exceeds 50*τ*. A double averaging of the numerical results for the absorbed power over a sufficiently long time interval Δ*t* and over a set of *N*_*exp*_ = 100 independent realizations of assemblies of *N*_*p*_ = 100 nanoparticles makes sure that the data obtained for the magnetic susceptibility of the assembly are statistically significant.

### Genetic algorithm implementation

To determine the volume fractions *λ*_*i*_ of basic assemblies in a linear combination of FMR spectra, which approximates the FMR spectrum of a dilute composite assembly with a given accuracy, a simple version of the genetic algorithm is used in this work. The genetic algorithm^34–36^ in a number of cases turns out to be very effective when choosing an optimal set from a large number of admissible data sets that satisfies certain, precisely formulated conditions. In this problem, the admissible data set is an arbitrary vector ***λ*** = {*λ*_*i*_}, *i* = 1–45, whose components are the volume fractions of the basic assemblies of nanoparticles in a certain composite assembly and obey the obvious relations

12 $$ 0 \le \lambda_{i} \le 1\;\;\;\;\;\;\;\;\;\;\sum\limits_{i = 1}^{45} {\lambda_{i} } = 1 $$

Each admissible vector ***λ*** according to Eq. (1) determines an approximate FMR spectrum, $\overline{\chi^{\prime\prime}}\left( j \right)$, *j* = 1 – *N*_*h*_, where *N*_*h*_ = 101 is the number of the external magnetic field values where the FMR spectra are specified. The approximate FMR spectrum can be compared with a given FMR spectrum by means of a fitness function

13 $$ \delta = {{\sum\limits_{j = 1}^{{N_{h} }} {\left| {\overline{\chi^{\prime\prime}}\left( j \right) - \overline{\chi^{\prime\prime}}^{(0)} \left( j \right)} \right|} } \mathord{\left/ {\vphantom {{\sum\limits_{j = 1}^{{N_{h} }} {\left| {\overline{\chi^{\prime\prime}}\left( j \right) - \overline{\chi^{\prime\prime}}^{(0)} \left( j \right)} \right|} } {\sum\limits_{j = 1}^{{N_{h} }} {\overline{\chi^{\prime\prime}}^{(0)} \left( j \right)} }}} \right. \kern-\nulldelimiterspace} {\sum\limits_{j = 1}^{{N_{h} }} {\overline{\chi^{\prime\prime}}^{(0)} \left( j \right)} }} $$

The problem is to choose the components of the vector ***λ*** in such a way that the residual *δ* does not exceed a sufficiently small value, say *δ* < ξ = 0.001, which means that the constructed optimal and specified FMR spectra are sufficiently close.

The structure of the genetic algorithm is not specified precisely^34,35^. It has to be chosen so as to ensure fast convergence of the algorithm iterations in a particular problem. In the implemented version of the genetic algorithm, at the first iteration, a set of 30 random vectors ***λ***_*s*_, *s* = 1–30 is created, and for each vector its residual *δ*_*s*_ is determined according to Eq. (13). Of the 30 initial vectors, 10 vectors are selected that have the smallest residuals *δ*_*s*_, the remaining vectors are discarded. The selected vectors are ordered according to the degree of residual increase, so that the vector ***λ***_1_ always has the least residual *δ*_1_ in the selected set of vectors.

The group of 10 vectors selected in such a way occupies the first 10 places in the formation of a new generation of vectors. The next group of 10 vectors in the new generation are formed from the vectors already selected using the well-known crossover technique^34,35^. Namely, when forming a vector of the second group, two vectors, ***λ***_*a*_ and ***λ***_*b*_, are arbitrarily selected from the vectors of the first group, and a random integer i* is assigned, such that 1 < i* < 45. The new vector ***λ***_*c*_ of the second group is constructed from parts of the vectors ***λ***_*a*_ and ***λ***_*b*_. Namely, its components are selected according to the following rule: ***λ***_*c*_(*i*) = ***λ***_*a*_(*i*) for components with numbers *i* ≤ i*, but ***λ***_*c*_(*i*) = ***λ***_*b*_(*i*) if i* < *i*. Then the components of the vector ***λ***_*c*_(*i*) are normalized to satisfy condition (12).

Thus, the vectors of the second group in the new generation are constructed using the crossover rocedure. Finally, 10 vectors of the third group in new generation are assigned randomly, taking into account the only condition, Eq. (12). As a result of these operations, a new generation of 30 vectors is created, and the whole procedure described above is repeated. Since the best vector of the previous generation is always included in the next generation, the smallest residual *δ*_1_ in the new generation certainly cannot exceed the previous one. As a rule, with subsequent iterations of the genetic algorithm the smallest residual decreases sequentially. Iterations stop when the condition *δ*_1_ < *ξ* is satisfied, where *ξ* is the assigned accuracy of determining the optimal vector ***λ***_1_.

## Supplementary Information

Supplementary Tables.

## Data Availability

The generated Supplementary Tables 1 and 2 for magnetic susceptibilities of assemblies of magnetite nanoparticles with magnetic damping constants *κ* = 0.1 and 0.2 are given in the Supplement Materials.
