# Supplementary material for: Deconvolution of ferromagnetic resonance spectrum of magnetic nanoparticle assembly using genetic algorithm
Source: Sci Rep. 2022 Feb 24;12:3126. doi: 10.1038/s41598-022-07105-7 (PMC8873211; doi:10.1038/s41598-022-07105-7)
Supplement: Supplementary file 1 — Supplementary Tables. [file 41598_2022_7105_MOESM1_ESM.doc]

Supplementary Information for the paper

Deconvolution of ferromagnetic resonance spectrum of magnetic nanoparticle assembly using genetic algorithm

By N. A. Usov1,2 & O. N. Serebryakova2

1National University of Science and Technology «MISiS», 119049, Moscow, Russia
2Pushkov Institute of Terrestrial Magnetism, Ionosphere and Radio Wave Propagation, Russian Academy of Sciences, IZMIRAN, 108480, Troitsk, Moscow, Russia

Table 1, magnetic damping constant  = 0.1


H	D = 5 nm
Oe	a/b = 1.0	D = 7 nm a/b = 1.0	D = 10 nm a/b = 1.0	D = 15 nm a/b = 1.0	D = 25 nm a/b = 1.0	D = 5 nm a/b = 1.1	D = 7 nm a/b = 1.1	D = 10 nm a/b = 1.1	D = 15 nm a/b = 1.1	D = 25 nm a/b = 1.1	D = 5 nm a/b = 1.2	D = 7 nm a/b = 1.2	D = 10 nm a/b = 1.2	D = 15 nm a/b = 1.2	D = 25 nm a/b = 1.2	D = 5 nm a/b = 1.3	D = 7 nm a/b = 1.3	D = 10 nm a/b = 1.3	D = 15 nm a/b = 1.3	D = 25 nm a/b = 1.3	D = 5 nm a/b = 1.4	D = 7 nm a/b = 1.4	D = 10 nm a/b = 1.4	D = 15 nm a/b = 1.4	D = 25 nm a/b = 1.4	D = 5 nm a/b = 1.5	D = 7 nm a/b = 1.5	D = 10 nm a/b = 1.5	D = 15 nm a/b = 1.5	D = 25 nm a/b = 1.5	D = 5 nm a/b = 1.6	D = 7 nm a/b = 1.6	D = 10 nm a/b = 1.6	D = 15 nm a/b = 1.6	D = 25 nm a/b = 1.6	D = 5 nm a/b = 1.7	D = 7 nm a/b = 1.7	D = 10 nm a/b = 1.7	D = 15 nm a/b = 1.7	D = 25 nm a/b = 1.7	D = 5 nm a/b = 1.8	D = 7 nm a/b = 1.8	D = 10 nm a/b = 1.8	D = 15 nm a/b = 1.8	D = 25 nm a/b = 1.8	
kap = 0.1	kap = 0.1	kap = 0.1	kap = 0.1	kap = 0.1	kap = 0.1	kap = 0.1	kap = 0.1	kap = 0.1	kap = 0.1	kap = 0.1	kap = 0.1	kap = 0.1	kap = 0.1	kap = 0.1	kap = 0.1	kap = 0.1	kap = 0.1	kap = 0.1	kap = 0.1	kap = 0.1	kap = 0.1	kap = 0.1	kap = 0.1	kap = 0.1	kap = 0.1	kap = 0.1	kap = 0.1	kap = 0.1	kap = 0.1	kap = 0.1	kap = 0.1	kap = 0.1	kap = 0.1	kap = 0.1	kap = 0.1	kap = 0.1	kap = 0.1	kap = 0.1	kap = 0.1	kap = 0.1	kap = 0.1	kap = 0.1	kap = 0.1	kap = 0.1	
0	0.01655	0.01408	0.01623	0.01686	0.02012	0.01607	0.01404	0.0154	0.0177	0.02083	0.01666	0.01733	0.0176	0.01867	0.02251	0.01927	0.01838	0.02087	0.02307	0.02675	0.01721	0.01789	0.02456	0.02918	0.03405	0.02134	0.02072	0.02694	0.03882	0.04274	0.02037	0.02551	0.03873	0.04941	0.05917	0.02676	0.02812	0.04653	0.06679	0.08347	0.02162	0.03037	0.06326	0.09712	0.12646	
40	0.01628	0.01337	0.0155	0.01684	0.02036	0.01574	0.01389	0.01555	0.01776	0.02103	0.01643	0.0172	0.01785	0.019	0.02262	0.01974	0.0185	0.02088	0.02306	0.02682	0.01707	0.01795	0.02454	0.02915	0.03386	0.0216	0.02083	0.02703	0.03857	0.04269	0.02063	0.02559	0.03836	0.04918	0.05872	0.02611	0.02788	0.04623	0.06565	0.08201	0.02104	0.03033	0.06284	0.09516	0.12424	
80	0.01606	0.01272	0.01507	0.01722	0.02102	0.01545	0.01413	0.01599	0.01821	0.02151	0.01625	0.01697	0.01804	0.01955	0.02303	0.02014	0.01874	0.02104	0.02342	0.02721	0.01691	0.01826	0.02485	0.02958	0.03405	0.0219	0.021	0.02754	0.03919	0.04335	0.02101	0.02572	0.03849	0.0499	0.05914	0.02547	0.02771	0.04675	0.06576	0.0822	0.0207	0.03078	0.06353	0.09537	0.12537	
120	0.01595	0.01244	0.01513	0.01802	0.022	0.01528	0.01487	0.0167	0.01906	0.02227	0.01619	0.01677	0.01819	0.02035	0.02381	0.02043	0.01911	0.02144	0.02422	0.02796	0.01679	0.01881	0.02553	0.03056	0.03471	0.02222	0.02124	0.02853	0.04077	0.04479	0.02154	0.02596	0.03927	0.05167	0.0607	0.02503	0.02772	0.04821	0.06755	0.08473	0.02072	0.03182	0.06558	0.09861	0.13084	
160	0.01598	0.01272	0.01577	0.01914	0.02318	0.01527	0.01609	0.01761	0.02027	0.02325	0.0163	0.01668	0.01838	0.02143	0.02496	0.02061	0.01962	0.02213	0.02547	0.02909	0.0168	0.0196	0.02658	0.03209	0.03593	0.02255	0.02158	0.03001	0.04325	0.04702	0.02224	0.0264	0.0408	0.05454	0.06366	0.02494	0.02804	0.05068	0.07143	0.09022	0.02122	0.03349	0.06918	0.10567	0.14139	
200	0.01613	0.01362	0.01691	0.02045	0.02437	0.01545	0.01767	0.01861	0.02178	0.02443	0.01656	0.01679	0.01871	0.02281	0.02644	0.0207	0.02026	0.02317	0.02716	0.03061	0.01701	0.02063	0.02795	0.03413	0.03778	0.0229	0.02209	0.032	0.0465	0.05001	0.02308	0.02716	0.04309	0.0585	0.06831	0.02528	0.02886	0.05418	0.07773	0.09924	0.0222	0.03574	0.07443	0.11713	0.15735	
240	0.01642	0.01501	0.01834	0.0218	0.02548	0.0158	0.01938	0.01961	0.02348	0.02575	0.01692	0.01712	0.01929	0.02447	0.02818	0.02078	0.021	0.02457	0.02924	0.0325	0.01746	0.02187	0.0296	0.03662	0.04026	0.02332	0.02286	0.03445	0.05035	0.05374	0.02404	0.02837	0.0461	0.06359	0.07496	0.02607	0.03033	0.05871	0.08676	0.11219	0.02362	0.03853	0.08141	0.13332	0.17858	
280	0.01682	0.01662	0.01984	0.02306	0.02646	0.01629	0.02095	0.02054	0.02528	0.02716	0.0173	0.01761	0.02019	0.02636	0.03008	0.0209	0.02182	0.0263	0.03163	0.03474	0.01818	0.02326	0.03147	0.03945	0.04335	0.02383	0.02395	0.03734	0.05462	0.05827	0.02506	0.03012	0.04979	0.06989	0.08393	0.02724	0.03259	0.06428	0.09876	0.12929	0.02536	0.04177	0.09017	0.15419	0.2045	
320	0.01731	0.01816	0.0212	0.02421	0.02739	0.01686	0.02216	0.02136	0.02711	0.02864	0.01764	0.01824	0.02143	0.02843	0.03207	0.02113	0.02268	0.02829	0.03425	0.03729	0.01911	0.02477	0.03354	0.04257	0.047	0.02446	0.0254	0.0406	0.05926	0.06373	0.02611	0.03247	0.05411	0.07761	0.09557	0.02871	0.03573	0.07095	0.1139	0.15051	0.02729	0.04541	0.10078	0.17933	0.23407	
360	0.01786	0.01937	0.02231	0.02531	0.02839	0.01748	0.02294	0.02214	0.0289	0.03018	0.01795	0.01894	0.02296	0.03059	0.03409	0.02154	0.02357	0.03047	0.03703	0.04009	0.02019	0.02633	0.03581	0.04594	0.05117	0.02522	0.02723	0.04417	0.06434	0.07042	0.02715	0.0354	0.0591	0.08704	0.11015	0.03036	0.03972	0.07885	0.13223	0.17553	0.02929	0.04945	0.11333	0.20795	0.26597	
400	0.01846	0.02018	0.02325	0.02648	0.02961	0.01814	0.02335	0.02297	0.03064	0.03178	0.01826	0.01972	0.02469	0.0328	0.03613	0.02217	0.02452	0.03276	0.03993	0.04308	0.02134	0.02789	0.03832	0.04957	0.05586	0.0261	0.02944	0.04802	0.07012	0.07872	0.02817	0.03884	0.06485	0.09857	0.12788	0.03208	0.0445	0.0882	0.15367	0.20375	0.03127	0.05397	0.12793	0.23892	0.2987	
440	0.01905	0.02067	0.02419	0.02789	0.03115	0.01884	0.02362	0.024	0.03232	0.03347	0.01867	0.02065	0.02654	0.03505	0.03824	0.02304	0.02555	0.03513	0.04295	0.04623	0.02248	0.02942	0.04115	0.05354	0.06116	0.02709	0.03199	0.05214	0.07703	0.08913	0.02918	0.04267	0.07158	0.11258	0.14879	0.03384	0.04993	0.09923	0.17795	0.23431	0.03325	0.0591	0.14467	0.27088	0.33072	
480	0.01963	0.02108	0.02536	0.02964	0.03306	0.0196	0.02404	0.02533	0.03399	0.03529	0.01928	0.02185	0.02848	0.03739	0.0405	0.02415	0.02674	0.0376	0.04612	0.04953	0.02359	0.03094	0.04437	0.05801	0.06725	0.02814	0.03489	0.05658	0.08559	0.10215	0.03019	0.04673	0.07957	0.12942	0.17269	0.03562	0.05586	0.11219	0.20459	0.26612	0.03531	0.06506	0.16357	0.30238	0.36064	
520	0.02016	0.02171	0.02695	0.03178	0.0353	0.02042	0.02491	0.02705	0.03568	0.03727	0.02019	0.02346	0.03053	0.0399	0.04301	0.02546	0.02817	0.04025	0.04951	0.05306	0.02469	0.03254	0.04808	0.06319	0.07448	0.02922	0.03812	0.06147	0.09634	0.11818	0.03123	0.05094	0.08913	0.14925	0.19916	0.0375	0.06217	0.12729	0.23287	0.298	0.03757	0.07208	0.1845	0.332	0.3873	
560	0.02066	0.02281	0.02906	0.03427	0.03774	0.02131	0.02641	0.02911	0.03749	0.03946	0.02146	0.02558	0.03281	0.04268	0.04584	0.0269	0.02992	0.0432	0.05324	0.05698	0.02585	0.03432	0.05233	0.06937	0.08328	0.03032	0.04173	0.06701	0.10971	0.13748	0.03233	0.05526	0.10057	0.17202	0.22753	0.03956	0.06883	0.1446	0.2619	0.3287	0.04016	0.08035	0.20716	0.35852	0.40987	
600	0.02114	0.02451	0.03159	0.03699	0.04028	0.02227	0.02854	0.03143	0.03951	0.04191	0.02305	0.02825	0.03548	0.04581	0.04905	0.02839	0.03205	0.04655	0.05747	0.06155	0.02719	0.03643	0.05718	0.07688	0.09418	0.03145	0.04575	0.07352	0.126	0.16002	0.0335	0.05974	0.11415	0.19737	0.2569	0.0419	0.07591	0.16401	0.29062	0.35708	0.04316	0.09005	0.23102	0.381	0.42786	
640	0.02168	0.02673	0.03433	0.03982	0.04285	0.02327	0.03113	0.03385	0.04184	0.04469	0.0249	0.0314	0.03869	0.04933	0.05267	0.02989	0.03457	0.05041	0.06237	0.06714	0.02881	0.03904	0.06269	0.08609	0.10769	0.03266	0.05023	0.08138	0.14522	0.1855	0.0348	0.06456	0.13001	0.22467	0.28624	0.04459	0.08358	0.18523	0.31797	0.38219	0.04658	0.10124	0.25534	0.39887	0.44115	
680	0.02239	0.02926	0.03703	0.04267	0.04552	0.02432	0.03389	0.03625	0.04461	0.04787	0.02691	0.03491	0.04255	0.05322	0.05674	0.03134	0.03748	0.05482	0.0682	0.07422	0.0308	0.04229	0.06897	0.0974	0.12423	0.03406	0.0552	0.09106	0.16712	0.21326	0.03629	0.07	0.14815	0.25304	0.31447	0.04767	0.0921	0.20779	0.34294	0.40332	0.05032	0.11388	0.27923	0.41197	0.44988	
720	0.02341	0.03179	0.03948	0.04559	0.04842	0.02547	0.03653	0.03856	0.0479	0.05151	0.02898	0.03858	0.04708	0.05744	0.06137	0.03278	0.04077	0.05978	0.07526	0.0833	0.0332	0.04627	0.07617	0.11118	0.14403	0.03577	0.0607	0.10298	0.19119	0.24242	0.0381	0.07639	0.16841	0.28143	0.34058	0.05114	0.10175	0.23103	0.36468	0.42007	0.05421	0.12781	0.30171	0.42048	0.45446	
760	0.02488	0.03411	0.04168	0.04871	0.05181	0.02679	0.03887	0.04086	0.05182	0.05569	0.03108	0.04225	0.05219	0.06198	0.06676	0.03428	0.04441	0.0653	0.08391	0.09492	0.03598	0.05105	0.08456	0.12774	0.16706	0.03796	0.06675	0.11753	0.21672	0.27189	0.04037	0.08407	0.19048	0.30873	0.36373	0.05497	0.11277	0.25419	0.38261	0.43235	0.05807	0.14277	0.32186	0.4249	0.45539	
800	0.02693	0.03616	0.04384	0.05226	0.05594	0.02846	0.04093	0.04333	0.05641	0.06049	0.03328	0.04584	0.05773	0.06693	0.07328	0.03598	0.04842	0.07142	0.0946	0.10953	0.03909	0.05666	0.0945	0.14727	0.19298	0.04077	0.07343	0.13493	0.24286	0.30051	0.04327	0.09334	0.21391	0.3339	0.38335	0.05908	0.12525	0.2765	0.39645	0.44033	0.06176	0.1584	0.33895	0.42587	0.4533	
840	0.02963	0.03814	0.04639	0.05647	0.06098	0.03064	0.04299	0.04628	0.06169	0.06596	0.0357	0.04938	0.06352	0.07257	0.08147	0.03806	0.05287	0.07831	0.10778	0.12749	0.04245	0.06309	0.10642	0.16978	0.22117	0.04431	0.08084	0.1552	0.26877	0.3272	0.04697	0.10438	0.23813	0.35606	0.39914	0.06342	0.13912	0.2972	0.40619	0.44442	0.06527	0.17429	0.35247	0.42412	0.44878	
880	0.03299	0.04048	0.04983	0.06152	0.06699	0.03352	0.04548	0.05003	0.06764	0.07224	0.03855	0.05305	0.0695	0.07938	0.09199	0.04071	0.05789	0.08632	0.12391	0.14895	0.04602	0.07042	0.12078	0.1951	0.25072	0.04864	0.08917	0.17809	0.29364	0.35106	0.05156	0.11725	0.2625	0.3746	0.41108	0.06792	0.15412	0.31566	0.41211	0.44512	0.06874	0.18999	0.36227	0.42032	0.44239	
920	0.03695	0.04368	0.05459	0.06744	0.07391	0.03723	0.04888	0.05486	0.0743	0.07952	0.042	0.05711	0.07577	0.08807	0.10558	0.04409	0.06369	0.09597	0.14334	0.17384	0.04983	0.07875	0.138	0.22277	0.28059	0.05372	0.09868	0.20308	0.31681	0.37143	0.05707	0.13188	0.28628	0.38921	0.41942	0.07257	0.16979	0.33137	0.4146	0.44304	0.0725	0.20506	0.36847	0.41503	0.43461	
960	0.04138	0.04821	0.06085	0.07411	0.08163	0.0418	0.05357	0.06091	0.08177	0.08812	0.0462	0.06182	0.08268	0.09954	0.12296	0.04829	0.07049	0.10795	0.16625	0.20183	0.05401	0.08829	0.1583	0.25212	0.30971	0.05945	0.10972	0.22938	0.33781	0.38803	0.06339	0.14801	0.30876	0.39986	0.42457	0.07738	0.18559	0.34403	0.41419	0.43873	0.07698	0.21908	0.37147	0.40863	0.42584	
1000	0.04612	0.05428	0.06844	0.08138	0.09014	0.04715	0.05963	0.0682	0.09032	0.09857	0.05116	0.06744	0.09085	0.11473	0.14467	0.05334	0.07853	0.12301	0.19259	0.23234	0.05876	0.09931	0.18172	0.28228	0.33706	0.06569	0.1226	0.25605	0.35633	0.40084	0.07032	0.1653	0.32922	0.40679	0.42701	0.08241	0.20095	0.35348	0.41137	0.43271	0.08265	0.23172	0.37182	0.40135	0.41638	
1040	0.05103	0.06179	0.07689	0.08921	0.09977	0.05307	0.06688	0.07665	0.10046	0.11162	0.0568	0.07409	0.10112	0.13447	0.17095	0.05921	0.08804	0.14185	0.222	0.26458	0.06436	0.11214	0.20794	0.31222	0.36186	0.07229	0.13761	0.28202	0.37226	0.41016	0.0776	0.18333	0.34699	0.41044	0.42727	0.08775	0.21537	0.35977	0.40662	0.42543	0.08992	0.24272	0.37009	0.39333	0.40647	
1080	0.05606	0.07033	0.08561	0.09785	0.11129	0.0593	0.07485	0.08625	0.11303	0.12825	0.06296	0.08184	0.11447	0.15936	0.20174	0.06584	0.09922	0.16494	0.25383	0.29759	0.07109	0.12708	0.23635	0.34088	0.38357	0.07916	0.15486	0.30631	0.3856	0.41649	0.08497	0.20162	0.36154	0.41134	0.42582	0.09355	0.22855	0.36308	0.40034	0.41727	0.09902	0.25195	0.36681	0.38463	0.39629	
1120	0.06129	0.07936	0.09422	0.10809	0.12611	0.0656	0.08308	0.09725	0.12916	0.14964	0.06949	0.09073	0.13186	0.18959	0.2366	0.07322	0.11229	0.19239	0.28713	0.3304	0.07922	0.14437	0.26601	0.36727	0.40193	0.08633	0.17427	0.32805	0.39643	0.42043	0.09232	0.21974	0.37251	0.41007	0.42303	0.10002	0.2404	0.36371	0.39286	0.40853	0.10993	0.25943	0.36236	0.37536	0.38599	
1160	0.06693	0.0885	0.1029	0.12137	0.14613	0.07181	0.09135	0.11034	0.15025	0.17705	0.07633	0.10096	0.15409	0.2249	0.27476	0.08142	0.12745	0.22385	0.32081	0.36205	0.08889	0.16413	0.2958	0.39057	0.41697	0.094	0.19549	0.34663	0.40491	0.42257	0.09971	0.23729	0.37976	0.40718	0.41922	0.1074	0.25107	0.36207	0.38452	0.3995	0.12241	0.26532	0.35696	0.36567	0.37572	
1200	0.07333	0.09787	0.11271	0.13975	0.17364	0.07798	0.10004	0.12675	0.1778	0.21163	0.0836	0.11297	0.18172	0.26455	0.31528	0.09064	0.14503	0.25853	0.35371	0.39175	0.10016	0.18628	0.32452	0.4102	0.42894	0.10254	0.21799	0.36175	0.41118	0.42346	0.10747	0.25398	0.38338	0.40315	0.41463	0.11601	0.26088	0.35864	0.37564	0.39043	0.13604	0.26991	0.35072	0.35583	0.36561	
1240	0.08095	0.10835	0.12578	0.16571	0.21087	0.08441	0.11031	0.14833	0.2132	0.25421	0.09169	0.12764	0.21494	0.30747	0.35715	0.10125	0.16543	0.29527	0.3848	0.41893	0.11297	0.21061	0.35105	0.42593	0.43821	0.11249	0.24107	0.37343	0.41543	0.42352	0.1162	0.26962	0.38375	0.39836	0.40943	0.12622	0.27029	0.35392	0.36659	0.38156	0.15029	0.2736	0.34369	0.34616	0.35581	
1280	0.09024	0.1217	0.14516	0.20178	0.25951	0.0916	0.12417	0.17727	0.25745	0.30509	0.10121	0.1462	0.25356	0.35236	0.39941	0.11369	0.18917	0.33271	0.41327	0.4433	0.12718	0.2368	0.37452	0.43785	0.44525	0.12444	0.264	0.38198	0.41784	0.42302	0.12666	0.28419	0.38149	0.39313	0.40382	0.13841	0.2798	0.34846	0.35777	0.37308	0.1647	0.27687	0.33595	0.33705	0.34648	
1320	0.10159	0.14043	0.17439	0.24998	0.32024	0.10024	0.14426	0.21583	0.31092	0.3638	0.11288	0.17024	0.29706	0.39788	0.44129	0.12845	0.2168	0.3695	0.43869	0.46488	0.1426	0.26446	0.39443	0.44638	0.45053	0.13898	0.28618	0.38791	0.41862	0.42213	0.13965	0.29776	0.37738	0.38769	0.39794	0.15289	0.28979	0.34277	0.34961	0.36514	0.17899	0.2802	0.3277	0.32884	0.33776	
1360	0.11516	0.16737	0.21685	0.31134	0.3923	0.11101	0.1734	0.26583	0.37304	0.42898	0.12744	0.20136	0.34466	0.44284	0.48219	0.14591	0.24884	0.40452	0.46102	0.48394	0.15905	0.2932	0.41073	0.45218	0.45449	0.15655	0.30724	0.39191	0.41803	0.42092	0.15584	0.3106	0.37231	0.38225	0.39196	0.16991	0.30054	0.3373	0.34248	0.35783	0.19309	0.28406	0.31928	0.32179	0.32979	
1400	0.13089	0.20509	0.27491	0.38535	0.47325	0.12445	0.21394	0.32809	0.44223	0.49836	0.1454	0.24085	0.39532	0.48623	0.52166	0.16622	0.28558	0.43703	0.48058	0.50094	0.17637	0.32271	0.42383	0.45606	0.45754	0.17731	0.32713	0.39469	0.41639	0.41942	0.17556	0.32305	0.36717	0.37699	0.38606	0.18947	0.31214	0.33245	0.33667	0.35116	0.20719	0.28879	0.31117	0.31604	0.32267	
1440	0.14839	0.25513	0.34912	0.4697	0.55906	0.14079	0.26709	0.40195	0.51584	0.56886	0.16686	0.28922	0.44786	0.52735	0.55929	0.18918	0.3269	0.46677	0.49795	0.5164	0.19442	0.35274	0.4345	0.45891	0.46005	0.20101	0.34613	0.39695	0.41408	0.41769	0.19864	0.33555	0.36274	0.37208	0.38038	0.21135	0.3245	0.32852	0.33236	0.34509	0.22161	0.29466	0.30394	0.31154	0.31644	
1480	0.16693	0.31735	0.43763	0.56028	0.64444	0.15979	0.33228	0.48498	0.5903	0.63682	0.19137	0.34587	0.50091	0.56563	0.5946	0.21416	0.37208	0.49388	0.51382	0.5308	0.21302	0.38308	0.44374	0.46154	0.46234	0.22702	0.36473	0.39927	0.41152	0.41584	0.22435	0.34854	0.35965	0.36771	0.37508	0.23499	0.33742	0.3257	0.32956	0.33955	0.2367	0.30175	0.29813	0.3081	0.3111	
1520	0.18557	0.38953	0.53593	0.6515	0.72344	0.18069	0.40682	0.57294	0.66145	0.69838	0.2179	0.40876	0.55293	0.60063	0.62687	0.24011	0.41971	0.51877	0.52879	0.54442	0.23192	0.41345	0.45261	0.46461	0.46472	0.25424	0.38351	0.40216	0.40917	0.41406	0.25137	0.36236	0.35828	0.36405	0.37027	0.25951	0.35062	0.32408	0.3281	0.33446	0.25267	0.31004	0.2942	0.30545	0.30661	
1560	0.20318	0.46727	0.63717	0.73685	0.79022	0.20226	0.48597	0.66007	0.72488	0.74983	0.24489	0.47446	0.60214	0.63179	0.65513	0.26561	0.46762	0.54189	0.54319	0.55729	0.25068	0.44343	0.46198	0.46857	0.46741	0.28126	0.40296	0.40596	0.40747	0.41256	0.27798	0.37721	0.35879	0.36127	0.36605	0.28371	0.36377	0.32369	0.32774	0.32979	0.26937	0.31937	0.29237	0.30324	0.30287	
1600	0.2186	0.54438	0.73289	0.80977	0.83983	0.22291	0.56333	0.7397	0.77648	0.78806	0.27043	0.53835	0.64654	0.65837	0.67812	0.28907	0.51304	0.56343	0.557	0.56908	0.26865	0.47229	0.47242	0.47356	0.47056	0.30645	0.42319	0.41087	0.40679	0.4116	0.30222	0.393	0.36111	0.35948	0.36252	0.30622	0.37658	0.3245	0.32817	0.32557	0.28627	0.32949	0.29262	0.30122	0.29978	
1640	0.23077	0.61367	0.81415	0.86447	0.86889	0.24094	0.63167	0.80504	0.81285	0.81091	0.29249	0.59513	0.68392	0.6794	0.6944	0.30885	0.55281	0.5832	0.56973	0.57914	0.28495	0.49898	0.48399	0.47944	0.47414	0.32819	0.44387	0.41693	0.4074	0.41135	0.32219	0.40935	0.36502	0.35876	0.35975	0.32558	0.38877	0.32646	0.32906	0.3219	0.30244	0.34008	0.29471	0.29921	0.29722	
1680	0.23885	0.66797	0.87286	0.8967	0.87601	0.25472	0.684	0.85018	0.83168	0.81735	0.30926	0.63954	0.71201	0.69369	0.70256	0.32355	0.58373	0.60044	0.58051	0.58658	0.29849	0.52207	0.49625	0.48576	0.47794	0.345	0.46409	0.42402	0.40935	0.41191	0.33633	0.42548	0.37015	0.35911	0.35782	0.34043	0.40005	0.32951	0.33016	0.31895	0.31662	0.35077	0.29816	0.2972	0.29513	
1720	0.24231	0.70127	0.90299	0.90433	0.86186	0.26301	0.71463	0.87087	0.83204	0.8076	0.31936	0.66709	0.72865	0.69996	0.70144	0.33212	0.60299	0.61387	0.58816	0.59032	0.30812	0.53992	0.50822	0.49183	0.48152	0.35575	0.48246	0.43181	0.41251	0.4132	0.34361	0.44031	0.37609	0.36044	0.35677	0.34965	0.41009	0.33358	0.33132	0.31694	0.32742	0.36113	0.30246	0.29534	0.29348	
1760	0.24099	0.70974	0.90157	0.88756	0.829	0.26507	0.72012	0.86516	0.81435	0.78303	0.32203	0.67479	0.73206	0.69711	0.69035	0.33401	0.60853	0.62184	0.59139	0.58933	0.31275	0.55078	0.51854	0.49678	0.48423	0.35976	0.49721	0.43977	0.4165	0.41494	0.3437	0.45254	0.38237	0.36255	0.35659	0.35249	0.41849	0.3386	0.33244	0.31603	0.33357	0.37068	0.30706	0.29389	0.29227	
1800	0.23508	0.69235	0.86912	0.84876	0.78134	0.26081	0.69984	0.83374	0.78038	0.74594	0.31725	0.6616	0.72113	0.68436	0.66926	0.32923	0.59941	0.62262	0.58899	0.5827	0.31155	0.55306	0.52564	0.49964	0.48526	0.35688	0.50647	0.44715	0.42072	0.41665	0.33695	0.46075	0.38848	0.36516	0.35718	0.3487	0.4247	0.34439	0.3335	0.31628	0.33414	0.37887	0.31156	0.29319	0.29154	
1840	0.22511	0.65106	0.8095	0.79207	0.72351	0.25079	0.65607	0.77974	0.7329	0.69923	0.30565	0.62866	0.6957	0.66155	0.63887	0.3183	0.57593	0.61472	0.58003	0.56988	0.30412	0.54561	0.52791	0.49941	0.48372	0.34745	0.50854	0.45302	0.42435	0.4177	0.3243	0.46363	0.39389	0.36787	0.35832	0.33851	0.4281	0.35071	0.33448	0.31763	0.32873	0.38509	0.31571	0.29352	0.29135	
1880	0.21183	0.59044	0.7293	0.72271	0.66014	0.23605	0.59362	0.70832	0.67535	0.64602	0.28839	0.57911	0.65665	0.62919	0.60059	0.30211	0.53969	0.59719	0.56402	0.55071	0.29059	0.52793	0.52398	0.49521	0.47877	0.33222	0.5022	0.45629	0.42648	0.41732	0.30709	0.46011	0.39801	0.37022	0.35964	0.32264	0.42803	0.35714	0.33529	0.3198	0.31754	0.38869	0.31941	0.29503	0.29172	
1920	0.19617	0.51695	0.6367	0.64622	0.59529	0.218	0.51903	0.62585	0.61142	0.58934	0.26692	0.51762	0.60598	0.58854	0.55638	0.28182	0.49334	0.5699	0.54101	0.5255	0.27166	0.50033	0.51293	0.48634	0.46976	0.31227	0.48692	0.45586	0.42615	0.41474	0.28684	0.44959	0.40022	0.37167	0.36061	0.30216	0.42383	0.36311	0.33578	0.32236	0.30135	0.389	0.32266	0.29772	0.29259	
1960	0.17911	0.43782	0.54025	0.56779	0.53203	0.19815	0.43951	0.53904	0.54468	0.5318	0.24282	0.44971	0.54663	0.54147	0.50849	0.25866	0.44031	0.53358	0.51157	0.49502	0.24853	0.46397	0.49436	0.47241	0.4563	0.28881	0.46294	0.4507	0.42249	0.40926	0.26498	0.43201	0.39985	0.37167	0.36063	0.2784	0.41499	0.3679	0.33567	0.32471	0.28138	0.38544	0.32549	0.30133	0.29384	
2000	0.16159	0.36	0.4476	0.49169	0.47232	0.17795	0.36189	0.45403	0.47827	0.47548	0.21754	0.38093	0.48216	0.49026	0.45922	0.2338	0.3843	0.48984	0.47675	0.46041	0.22277	0.42077	0.46854	0.45334	0.43835	0.26311	0.43133	0.44004	0.41479	0.40036	0.2427	0.40788	0.39628	0.36964	0.359	0.25278	0.40126	0.37068	0.33458	0.32617	0.25911	0.37756	0.32782	0.30538	0.29519	
2040	0.14446	0.28913	0.36462	0.421	0.41707	0.15855	0.29166	0.37573	0.41469	0.4218	0.19228	0.31613	0.41639	0.43735	0.41065	0.20828	0.32888	0.44094	0.43795	0.42301	0.1961	0.3732	0.43638	0.42942	0.41624	0.23634	0.39382	0.42348	0.40265	0.38774	0.22082	0.37827	0.38895	0.36509	0.35507	0.22664	0.38269	0.37058	0.33209	0.32607	0.23599	0.36515	0.32942	0.30923	0.29628	
2080	0.12836	0.22898	0.29481	0.35751	0.36641	0.14078	0.23238	0.30736	0.35573	0.37165	0.16796	0.25891	0.35291	0.38502	0.36438	0.18296	0.27706	0.3895	0.39676	0.38428	0.17016	0.32399	0.3993	0.4013	0.39063	0.2095	0.35254	0.40107	0.38598	0.37142	0.19982	0.34467	0.37749	0.35762	0.34832	0.20118	0.35969	0.36684	0.32779	0.3238	0.21326	0.34829	0.32982	0.31211	0.29666	
2120	0.11377	0.18116	0.23928	0.30194	0.32003	0.12508	0.18548	0.25042	0.30249	0.32543	0.14523	0.21133	0.2947	0.33519	0.32149	0.15851	0.231	0.33814	0.35479	0.3456	0.14631	0.27579	0.35908	0.36993	0.36243	0.18343	0.30984	0.37339	0.36506	0.3517	0.17985	0.30881	0.36179	0.34697	0.33843	0.17732	0.333	0.35887	0.32135	0.31895	0.19179	0.32742	0.32842	0.31328	0.29583	
2160	0.10092	0.14537	0.19711	0.25416	0.27748	0.11156	0.15048	0.20479	0.25549	0.28322	0.12449	0.17388	0.24384	0.28927	0.28251	0.13549	0.19192	0.28919	0.31353	0.30813	0.12546	0.23087	0.3177	0.33645	0.33266	0.15877	0.26791	0.3415	0.34052	0.32914	0.1609	0.27246	0.34205	0.33307	0.32531	0.15572	0.30367	0.34638	0.31256	0.31127	0.17203	0.30328	0.32449	0.31204	0.29331	
2200	0.08987	0.11984	0.166	0.21355	0.23846	0.10006	0.12548	0.16924	0.21479	0.24498	0.10604	0.14576	0.20135	0.24805	0.24748	0.11436	0.16007	0.24444	0.27421	0.27278	0.10801	0.19092	0.27703	0.3021	0.30236	0.13601	0.2286	0.30682	0.31324	0.30451	0.14288	0.23724	0.31877	0.31604	0.30917	0.13672	0.27286	0.32943	0.30137	0.30078	0.15406	0.27688	0.31737	0.30789	0.2887	
2240	0.08048	0.10206	0.14302	0.1793	0.20291	0.09031	0.10779	0.14188	0.18014	0.21059	0.09006	0.1253	0.16723	0.21183	0.21616	0.09551	0.13495	0.20498	0.23777	0.24011	0.09391	0.15691	0.23874	0.26808	0.27244	0.1155	0.19327	0.27099	0.28427	0.27867	0.12577	0.20445	0.29278	0.29623	0.29047	0.1204	0.24178	0.30843	0.28791	0.2877	0.13768	0.24938	0.30656	0.30057	0.28173	
2280	0.07249	0.0894	0.12531	0.15058	0.171	0.08199	0.09468	0.12066	0.1511	0.17996	0.07667	0.11045	0.14067	0.18048	0.18813	0.07929	0.11552	0.17123	0.2048	0.21042	0.0827	0.12907	0.20405	0.23547	0.24366	0.09746	0.1627	0.23567	0.25473	0.25248	0.10966	0.17499	0.26508	0.27414	0.26984	0.10665	0.21156	0.28413	0.27242	0.27239	0.12259	0.22196	0.29188	0.29002	0.27228	
2320	0.06556	0.07967	0.1106	0.12672	0.14302	0.07477	0.08386	0.10381	0.12713	0.15303	0.06592	0.09921	0.12032	0.15361	0.163	0.06594	0.10054	0.14304	0.1756	0.18374	0.07372	0.10705	0.17372	0.20512	0.21654	0.08203	0.13711	0.20235	0.22566	0.22675	0.09475	0.14929	0.23679	0.25047	0.24805	0.09521	0.18311	0.25753	0.25527	0.25535	0.1085	0.19569	0.27351	0.27648	0.26044	
2360	0.05934	0.07141	0.09745	0.10713	0.11919	0.06842	0.07382	0.08998	0.10764	0.12975	0.05773	0.08998	0.1046	0.13073	0.14047	0.05557	0.08876	0.11987	0.1502	0.15996	0.06625	0.09005	0.148	0.17761	0.19139	0.06923	0.11629	0.1722	0.19796	0.20214	0.08132	0.12741	0.20898	0.22597	0.22585	0.08571	0.15711	0.22978	0.23688	0.23711	0.09526	0.17137	0.25201	0.26035	0.24646	
2400	0.05349	0.06389	0.08523	0.09129	0.09958	0.06276	0.0639	0.07836	0.09202	0.11	0.05188	0.0817	0.09205	0.11136	0.1204	0.04808	0.07913	0.10095	0.12843	0.13885	0.05967	0.07706	0.12668	0.15321	0.16835	0.05899	0.0997	0.14595	0.1723	0.17909	0.06963	0.10909	0.18257	0.20146	0.20392	0.07778	0.13395	0.202	0.21769	0.21822	0.08289	0.14955	0.2283	0.24219	0.23075	
2440	0.04779	0.05703	0.07394	0.0787	0.08396	0.05764	0.05416	0.06854	0.07962	0.09361	0.04794	0.07385	0.08147	0.09507	0.10276	0.04319	0.07089	0.08553	0.10998	0.1202	0.05355	0.06705	0.10925	0.13197	0.14743	0.0511	0.08661	0.12388	0.14911	0.15791	0.05987	0.09388	0.15825	0.17769	0.18282	0.07102	0.11379	0.17526	0.19811	0.19913	0.07155	0.1304	0.20347	0.22264	0.21381	
2480	0.04212	0.05106	0.06389	0.06881	0.07184	0.05295	0.04507	0.06039	0.06983	0.08029	0.04532	0.06635	0.07209	0.08152	0.08755	0.04038	0.0636	0.07294	0.09448	0.1038	0.04768	0.05912	0.09498	0.11372	0.12859	0.04526	0.07627	0.10587	0.12857	0.13869	0.05209	0.08124	0.13641	0.15533	0.16295	0.06509	0.09658	0.15038	0.17854	0.18028	0.06149	0.11384	0.17869	0.20236	0.19617	
2520	0.03648	0.04626	0.0554	0.06103	0.06254	0.04861	0.03723	0.0538	0.06204	0.06963	0.04337	0.05933	0.06353	0.07039	0.07478	0.03901	0.05705	0.06267	0.08152	0.08948	0.04206	0.05258	0.08311	0.09815	0.11174	0.04109	0.06795	0.09145	0.11068	0.12143	0.04617	0.07069	0.1172	0.13491	0.14451	0.0597	0.08215	0.12797	0.15935	0.16198	0.05293	0.09956	0.15501	0.18196	0.17837	
2560	0.03101	0.04274	0.04861	0.05475	0.05531	0.04451	0.03111	0.04864	0.0557	0.06116	0.04141	0.05299	0.05571	0.06139	0.06435	0.03836	0.05116	0.05437	0.07071	0.07711	0.03682	0.04699	0.07294	0.0849	0.09682	0.03815	0.06105	0.07998	0.09531	0.10601	0.04185	0.06181	0.10052	0.11675	0.12761	0.05466	0.07026	0.10837	0.14087	0.1445	0.04597	0.08717	0.13329	0.16199	0.16088	
2600	0.02591	0.04032	0.04342	0.04947	0.04947	0.04059	0.02686	0.04463	0.05034	0.05438	0.03893	0.04744	0.04873	0.05417	0.05603	0.03778	0.04593	0.04781	0.06168	0.06657	0.03217	0.04211	0.06396	0.07361	0.08375	0.03601	0.05512	0.07076	0.08223	0.09231	0.03878	0.05431	0.08615	0.101	0.11223	0.04983	0.06062	0.09165	0.12341	0.12803	0.04055	0.07624	0.11406	0.14289	0.14408	
2640	0.02143	0.03862	0.03947	0.04477	0.04449	0.03681	0.02434	0.04137	0.04562	0.04881	0.03562	0.04266	0.04272	0.04837	0.0495	0.03675	0.04134	0.04276	0.05411	0.05771	0.02827	0.03783	0.05585	0.06394	0.07244	0.03429	0.04984	0.06316	0.07118	0.08018	0.03659	0.04801	0.0738	0.08761	0.09834	0.04517	0.05295	0.07773	0.10725	0.11274	0.03647	0.06647	0.09754	0.12499	0.12824	
2680	0.01779	0.03713	0.03634	0.04043	0.04006	0.03318	0.02313	0.03845	0.04132	0.04407	0.03146	0.03849	0.03775	0.04358	0.04434	0.03499	0.03732	0.039	0.04774	0.05037	0.0252	0.03411	0.0485	0.05564	0.06279	0.03269	0.045	0.05665	0.06192	0.06953	0.03493	0.04279	0.0632	0.07639	0.08588	0.04071	0.04695	0.06637	0.0926	0.09871	0.03338	0.05766	0.08368	0.10852	0.11354	
2720	0.01512	0.03538	0.0336	0.03637	0.03608	0.02974	0.02272	0.03552	0.03734	0.03986	0.02671	0.03473	0.03376	0.03945	0.04013	0.03247	0.03375	0.03626	0.04236	0.04435	0.02294	0.03093	0.04193	0.0485	0.05465	0.03104	0.04051	0.05092	0.0542	0.06025	0.03355	0.03856	0.0541	0.06703	0.07478	0.03652	0.04234	0.05723	0.07963	0.08603	0.0309	0.04973	0.07219	0.09361	0.10004	
2760	0.01349	0.03312	0.031	0.03264	0.03257	0.0266	0.02262	0.03236	0.03369	0.03601	0.02184	0.03121	0.03059	0.03568	0.03653	0.02935	0.03051	0.03423	0.03779	0.03941	0.02138	0.02824	0.03627	0.04236	0.04786	0.02927	0.03635	0.04577	0.04783	0.05227	0.03229	0.03523	0.04636	0.05919	0.06501	0.03269	0.03881	0.04996	0.06839	0.07473	0.02871	0.04272	0.0627	0.08031	0.08776	
2800	0.0128	0.03032	0.02844	0.02934	0.0296	0.02386	0.02247	0.02896	0.03041	0.03245	0.01741	0.02783	0.02801	0.03211	0.03325	0.02598	0.02749	0.03256	0.03391	0.03531	0.02033	0.02598	0.03163	0.0371	0.04221	0.02742	0.03256	0.04115	0.0426	0.04551	0.03105	0.03268	0.03985	0.05253	0.05652	0.02932	0.03606	0.04421	0.05885	0.0648	0.02659	0.03672	0.0548	0.06862	0.07665	
2840	0.0129	0.02718	0.02599	0.02652	0.02723	0.02161	0.02208	0.02547	0.02759	0.02916	0.01392	0.02461	0.02579	0.02868	0.03018	0.02273	0.02464	0.03097	0.03062	0.03184	0.0196	0.02405	0.02806	0.03263	0.03749	0.02558	0.02917	0.03707	0.03832	0.03988	0.02981	0.03075	0.03448	0.04673	0.04926	0.02646	0.03377	0.03966	0.05089	0.05619	0.02445	0.03179	0.0481	0.05848	0.06667	
2880	0.01353	0.02401	0.02382	0.02419	0.02541	0.0199	0.02142	0.02214	0.02526	0.02618	0.01171	0.02167	0.02377	0.02548	0.02727	0.01991	0.02199	0.02923	0.02782	0.02882	0.01901	0.02238	0.02554	0.02886	0.03351	0.02385	0.02626	0.03353	0.03481	0.03526	0.02854	0.02925	0.03017	0.04157	0.04314	0.02412	0.03169	0.036	0.04431	0.04882	0.02237	0.02795	0.04235	0.04981	0.05777	
2920	0.01443	0.02117	0.02206	0.02225	0.02401	0.01871	0.02055	0.01923	0.02341	0.02354	0.0109	0.01914	0.02186	0.02261	0.02458	0.01772	0.01958	0.02724	0.02544	0.02613	0.01844	0.02091	0.02391	0.02571	0.0301	0.02231	0.02384	0.03053	0.03189	0.03152	0.02719	0.02799	0.02678	0.03692	0.03805	0.02226	0.0296	0.03297	0.03886	0.04259	0.0205	0.02512	0.03736	0.04249	0.0499	
2960	0.01534	0.01886	0.02073	0.02054	0.02286	0.01795	0.01955	0.01695	0.02196	0.02127	0.01131	0.01714	0.02004	0.02023	0.02217	0.01618	0.01749	0.02501	0.02339	0.02369	0.01782	0.01962	0.02296	0.0231	0.02716	0.021	0.02192	0.02802	0.02939	0.02848	0.02569	0.02679	0.02414	0.03273	0.03383	0.0208	0.02736	0.03036	0.0343	0.03737	0.01904	0.02314	0.03303	0.03642	0.04304	
3000	0.01607	0.01716	0.01975	0.01888	0.02177	0.0175	0.01848	0.01534	0.02079	0.01935	0.01258	0.01568	0.01837	0.0184	0.02012	0.01516	0.01579	0.02262	0.02161	0.0215	0.01712	0.01848	0.0224	0.02097	0.0246	0.0199	0.02042	0.0259	0.02719	0.026	0.02395	0.02551	0.02206	0.029	0.03033	0.01964	0.02494	0.02797	0.03042	0.03303	0.01813	0.02179	0.0293	0.03145	0.03719	
3040	0.01653	0.01595	0.01894	0.01715	0.0206	0.01722	0.01733	0.01435	0.01975	0.01774	0.01419	0.0147	0.0169	0.01712	0.01843	0.01442	0.01449	0.02024	0.02001	0.01954	0.01635	0.0175	0.02198	0.01927	0.0224	0.01894	0.01926	0.02405	0.02519	0.0239	0.02191	0.02409	0.02035	0.02578	0.02737	0.01867	0.02237	0.02569	0.02707	0.02944	0.01781	0.02081	0.02616	0.02746	0.0323	
3080	0.01668	0.01502	0.01808	0.0153	0.01929	0.01698	0.01607	0.01378	0.01872	0.0164	0.01564	0.01405	0.01567	0.01627	0.01706	0.0137	0.01353	0.01801	0.01854	0.01783	0.01552	0.01665	0.02147	0.01793	0.02052	0.01804	0.01829	0.02234	0.02333	0.02206	0.01953	0.02249	0.01886	0.02311	0.02483	0.01781	0.01976	0.02343	0.02416	0.02646	0.01801	0.01999	0.02353	0.02429	0.02834	
3120	0.01657	0.01416	0.01698	0.01342	0.01788	0.01668	0.01468	0.0134	0.01757	0.01527	0.01653	0.01353	0.01466	0.01568	0.01594	0.01279	0.01281	0.01608	0.01716	0.01637	0.01462	0.01589	0.02073	0.01691	0.01895	0.01712	0.01739	0.0207	0.02161	0.02039	0.01687	0.02075	0.01745	0.02099	0.02258	0.01699	0.01727	0.02117	0.02165	0.02398	0.01853	0.01916	0.02136	0.02181	0.02523	
3160	0.01628	0.01322	0.01558	0.01165	0.01645	0.01628	0.01318	0.01297	0.01628	0.0143	0.01661	0.01298	0.0138	0.01514	0.01496	0.01158	0.01218	0.01452	0.01584	0.01513	0.01367	0.01516	0.01968	0.01614	0.01764	0.01614	0.01642	0.01904	0.02002	0.01882	0.01404	0.01894	0.01609	0.01937	0.02056	0.01619	0.01505	0.01892	0.01951	0.02189	0.01914	0.01823	0.01955	0.01986	0.02285	
3200	0.01588	0.0122	0.01391	0.01018	0.01512	0.0158	0.01166	0.01237	0.01485	0.01345	0.01584	0.0123	0.01301	0.01449	0.01403	0.01012	0.01152	0.01333	0.01458	0.01407	0.01264	0.01436	0.01836	0.01554	0.01652	0.01507	0.01534	0.01738	0.01857	0.01731	0.01124	0.01713	0.01478	0.01817	0.01873	0.0154	0.01322	0.01673	0.01776	0.0201	0.01955	0.01716	0.018	0.0183	0.02104	
3240	0.01545	0.01122	0.01215	0.00916	0.01396	0.01526	0.01026	0.01153	0.01333	0.01267	0.01438	0.01148	0.01221	0.01361	0.01311	0.00853	0.01074	0.01249	0.01341	0.01312	0.01155	0.01344	0.01684	0.01501	0.01553	0.01397	0.01414	0.01574	0.01725	0.01586	0.00868	0.01541	0.01356	0.01724	0.01709	0.01462	0.01184	0.01468	0.01635	0.01851	0.01957	0.01598	0.0166	0.01702	0.01963	
3280	0.015	0.01047	0.01048	0.00865	0.013	0.01472	0.00916	0.01053	0.0118	0.01196	0.01247	0.01062	0.01135	0.01252	0.01216	0.00707	0.0098	0.01192	0.01236	0.01223	0.01041	0.01237	0.01522	0.01447	0.01458	0.0129	0.01288	0.01416	0.01603	0.01448	0.00658	0.01387	0.01247	0.01644	0.01563	0.01385	0.01092	0.01283	0.01522	0.01704	0.01907	0.01474	0.0153	0.01591	0.01843	
3320	0.01453	0.01013	0.00907	0.00862	0.01218	0.01419	0.0085	0.00947	0.01037	0.0113	0.01042	0.00983	0.01044	0.0113	0.01122	0.00595	0.00873	0.01152	0.01146	0.01136	0.00928	0.01115	0.01361	0.01382	0.01362	0.01193	0.01169	0.01271	0.01489	0.01318	0.00506	0.01255	0.01155	0.01562	0.01436	0.01308	0.0104	0.01124	0.01428	0.01567	0.01802	0.01351	0.01405	0.01489	0.01731	
3360	0.01401	0.01029	0.00801	0.00889	0.01142	0.01366	0.00833	0.00852	0.00911	0.01068	0.00848	0.00924	0.00954	0.01009	0.01032	0.00535	0.00762	0.01121	0.01075	0.01049	0.00823	0.00986	0.0121	0.013	0.0126	0.01112	0.01068	0.01144	0.01376	0.01199	0.00418	0.0115	0.01081	0.0147	0.01327	0.01226	0.01017	0.00994	0.01345	0.01434	0.01652	0.01233	0.01288	0.01392	0.01614	
3400	0.01339	0.01086	0.00726	0.00924	0.01061	0.0131	0.0086	0.00779	0.00807	0.01008	0.00686	0.0089	0.00874	0.00905	0.00951	0.00534	0.00657	0.01094	0.01022	0.00964	0.00732	0.00862	0.01076	0.01199	0.01154	0.01049	0.00997	0.01039	0.01261	0.01093	0.00389	0.0107	0.01022	0.01366	0.01235	0.01135	0.01012	0.00895	0.01264	0.01309	0.01471	0.01121	0.01181	0.013	0.01491	
3440	0.01266	0.01165	0.0067	0.00943	0.00968	0.01243	0.00916	0.00734	0.00728	0.00952	0.00561	0.00882	0.0081	0.00828	0.00882	0.00587	0.0057	0.01063	0.00986	0.00882	0.00661	0.00753	0.0096	0.01081	0.01047	0.01003	0.00961	0.00958	0.01145	0.01004	0.00407	0.01013	0.00973	0.01252	0.01159	0.01031	0.01012	0.00824	0.01182	0.01193	0.0128	0.01018	0.01091	0.01215	0.01363	
3480	0.01182	0.01236	0.00613	0.0093	0.00863	0.01159	0.00979	0.00716	0.00675	0.00897	0.00473	0.0089	0.00767	0.00783	0.00827	0.00678	0.00508	0.01027	0.00965	0.00807	0.00615	0.0067	0.00863	0.00954	0.00943	0.00967	0.00959	0.00898	0.01029	0.00932	0.00452	0.00974	0.00929	0.01138	0.01096	0.00913	0.01006	0.00779	0.01099	0.01091	0.01098	0.00922	0.01021	0.01142	0.01241	
3520	0.01091	0.01268	0.00543	0.00878	0.00751	0.01056	0.01027	0.00719	0.00642	0.00846	0.00416	0.00901	0.00744	0.00765	0.00784	0.00785	0.00472	0.00982	0.00953	0.00744	0.00595	0.00619	0.00785	0.00827	0.00849	0.00934	0.00981	0.00858	0.00919	0.00881	0.00507	0.00946	0.00887	0.01033	0.01044	0.00785	0.00989	0.00754	0.01021	0.01007	0.00938	0.00834	0.00976	0.01084	0.01136	
3560	0.01002	0.01242	0.00451	0.00792	0.00643	0.00935	0.01043	0.0073	0.00627	0.00796	0.00381	0.00899	0.00731	0.00764	0.00748	0.00883	0.00461	0.00928	0.00947	0.00694	0.00595	0.00601	0.00723	0.00712	0.0077	0.00895	0.01013	0.00833	0.00823	0.00849	0.00555	0.00922	0.00845	0.00949	0.00999	0.00653	0.00957	0.00744	0.00955	0.00945	0.00813	0.00754	0.00955	0.01045	0.01059	
3600	0.00921	0.01154	0.00342	0.00686	0.00552	0.00802	0.01021	0.00741	0.00622	0.0075	0.00363	0.00877	0.00719	0.00766	0.00717	0.00953	0.00469	0.00865	0.0094	0.00655	0.00611	0.00608	0.00675	0.00617	0.00708	0.00842	0.01039	0.00818	0.00746	0.00834	0.00586	0.00895	0.00803	0.00889	0.00958	0.0053	0.0091	0.00742	0.00907	0.00904	0.00725	0.00684	0.00953	0.01024	0.01016	
3640	0.00855	0.01019	0.00233	0.0058	0.00485	0.00668	0.00966	0.00741	0.00622	0.00705	0.00358	0.00831	0.00698	0.00756	0.00686	0.00984	0.00486	0.00792	0.00928	0.00625	0.00636	0.00631	0.00639	0.00548	0.00663	0.00773	0.01046	0.00807	0.00691	0.00831	0.00597	0.00861	0.00764	0.00852	0.00917	0.00428	0.00853	0.00742	0.0088	0.00877	0.00675	0.00625	0.00962	0.01015	0.01001	
3680	0.0081	0.00865	0.0014	0.00489	0.00444	0.00545	0.00889	0.00725	0.0062	0.00662	0.00367	0.00768	0.00659	0.00726	0.00654	0.00975	0.00505	0.00714	0.0091	0.00598	0.00665	0.00657	0.00611	0.00503	0.00631	0.00689	0.01025	0.00795	0.00655	0.00831	0.00591	0.00819	0.0073	0.00829	0.00869	0.00358	0.0079	0.00734	0.00869	0.00855	0.00656	0.00577	0.0097	0.01006	0.00999	
3720	0.00784	0.00725	8.2403E-4	0.00424	0.00423	0.00442	0.00807	0.00693	0.0061	0.00617	0.0039	0.00701	0.00602	0.00672	0.0062	0.00934	0.00519	0.00634	0.00882	0.00569	0.00692	0.00674	0.00588	0.00477	0.00607	0.00594	0.00975	0.00776	0.00628	0.00825	0.00574	0.00767	0.00702	0.00805	0.00808	0.00327	0.00725	0.00711	0.00862	0.00821	0.00661	0.00537	0.00963	0.00982	0.00982	
3760	0.00775	0.00628	6.69416E-4	0.00384	0.00409	0.00365	0.00733	0.00646	0.00587	0.00569	0.00426	0.00644	0.00532	0.006	0.00582	0.00875	0.00525	0.00555	0.00845	0.00534	0.00715	0.00675	0.00564	0.0046	0.00585	0.00496	0.00901	0.00747	0.00599	0.00802	0.00554	0.00712	0.00677	0.00765	0.00727	0.00333	0.00664	0.00666	0.00843	0.0076	0.00679	0.00503	0.00928	0.00921	0.00916	
3800	0.00777	0.00586	8.92408E-4	0.00363	0.0039	0.00316	0.00675	0.00588	0.00548	0.00517	0.00473	0.00607	0.00459	0.00517	0.00541	0.00814	0.00522	0.00484	0.00797	0.00489	0.00735	0.00656	0.00536	0.00441	0.00558	0.00404	0.00813	0.00705	0.00553	0.00754	0.00536	0.00657	0.0065	0.00693	0.00623	0.0037	0.00611	0.00592	0.00794	0.00656	0.00701	0.00467	0.00854	0.00805	0.00766	
3840	0.00781	0.00596	0.00133	0.00346	0.00355	0.00289	0.00632	0.00522	0.00494	0.00462	0.00524	0.00596	0.00396	0.00434	0.00499	0.00766	0.00511	0.00422	0.0074	0.00435	0.00753	0.00618	0.00501	0.00412	0.00522	0.00327	0.00726	0.00651	0.00478	0.00678	0.00525	0.00607	0.00614	0.00583	0.00493	0.00423	0.00566	0.00487	0.007	0.00497	0.00717	0.00424	0.00737	0.00619	0.00504	
3880	0.00782	0.00639	0.00175	0.00323	0.003	0.00275	0.00597	0.00455	0.00427	0.00406	0.0057	0.00609	0.00353	0.00362	0.00455	0.00741	0.00497	0.00372	0.00676	0.00375	0.00767	0.00569	0.00456	0.00367	0.00476	0.0027	0.0065	0.00587	0.00371	0.00574	0.00521	0.00569	0.00563	0.00436	0.00341	0.00476	0.00529	0.00355	0.00554	0.00279	0.00716	0.00371	0.0058	0.00356	0.00121	
3920	0.00774	0.00686	0.00194	0.00287	0.00232	0.00266	0.00562	0.00392	0.00355	0.00352	0.00602	0.00635	0.00333	0.00308	0.00411	0.00742	0.00482	0.00333	0.00611	0.00314	0.00779	0.00516	0.00405	0.00308	0.00422	0.00235	0.00595	0.0052	0.00237	0.00455	0.00522	0.00542	0.00496	0.00264	0.00175	0.00513	0.005	0.00205	0.00359	1.11234E-4	0.00696	0.00308	0.00395	2.18865E-4	0.00111	
3960	0.00757	0.00711	0.00177	0.0024	0.00164	0.00252	0.00521	0.00339	0.00286	0.00308	0.00616	0.00662	0.00337	0.00276	0.00372	0.00767	0.0047	0.00305	0.00548	0.00259	0.00786	0.00469	0.00352	0.00242	0.00363	0.00222	0.00561	0.00457	9.12665E-4	0.00333	0.00529	0.00528	0.00417	8.90497E-4	7.84799E-5	0.00522	0.00474	5.34529E-4	0.00133	8.82389E-4	0.00654	0.00241	0.00201	5.82973E-4	1.11451E-4	
4000	0.00732	0.00698	0.00123	0.00193	0.00112	0.0023	0.00476	0.00302	0.00232	0.0028	0.00612	0.0068	0.00357	0.00265	0.00341	0.00808	0.00463	0.00285	0.00497	0.00218	0.00786	0.00434	0.00307	0.00182	0.00307	0.00227	0.00548	0.00408	4.12613E-4	0.0023	0.00539	0.00524	0.00335	6.28523E-4	4.0756E-4	0.00499	0.0045	4.02434E-4	9.50609E-4	5.57368E-4	0.00595	0.0018	2.54117E-4	4.26465E-4	7.45134E-4	

Table 2, magnetic damping constant  = 0.2


H	D = 5 nm	D = 7 nm	D = 10 nm	D = 15 nm	D = 25 nm	D = 5 nm	D = 7 nm	D = 10 nm	D = 15 nm	D = 25 nm	D = 5 nm	D = 7 nm	D = 10 nm	D = 15 nm	D = 25 nm	D = 5 nm	D = 7 nm	D = 10 nm	D = 15 nm	D = 25 nm	D = 5 nm	D = 7 nm	D = 10 nm	D = 15 nm	D = 25 nm	D = 5 nm	D = 7 nm	D = 10 nm	D = 15 nm	D = 25 nm	D = 5 nm	D = 7 nm	D = 10 nm	D = 15 nm	D = 25 nm	D = 5 nm	D = 7 nm	D = 10 nm	D = 15 nm	D = 25 nm	D = 5 nm	D = 7 nm	D = 10 nm	D = 15 nm	D = 25 nm	
Oe	a/b = 1.0	a/b = 1.0	a/b = 1.0	a/b = 1.0	a/b = 1.0	a/b = 1.1	a/b = 1.1	a/b = 1.1	a/b = 1.1	a/b = 1.1	a/b = 1.2	a/b = 1.2	a/b = 1.2	a/b = 1.2	a/b = 1.2	a/b = 1.3	a/b = 1.3	a/b = 1.3	a/b = 1.3	a/b = 1.3	a/b = 1.4	a/b = 1.4	a/b = 1.4	a/b = 1.4	a/b = 1.4	a/b = 1.5	a/b = 1.5	a/b = 1.5	a/b = 1.5	a/b = 1.5	a/b = 1.6	a/b = 1.6	a/b = 1.6	a/b = 1.6	a/b = 1.6	a/b = 1.7	a/b = 1.7	a/b = 1.7	a/b = 1.7	a/b = 1.7	a/b = 1.8	a/b = 1.8	a/b = 1.8	a/b = 1.8	a/b = 1.8	
kap = 0.2	kap = 0.2	kap = 0.2	kap = 0.2	kap = 0.2	kap = 0.2	kap = 0.2	kap = 0.2	kap = 0.2	kap = 0.2	kap = 0.2	kap = 0.2	kap = 0.2	kap = 0.2	kap = 0.2	kap = 0.2	kap = 0.2	kap = 0.2	kap = 0.2	kap = 0.2	kap = 0.2	kap = 0.2	kap = 0.2	kap = 0.2	kap = 0.2	kap = 0.2	kap = 0.2	kap = 0.2	kap = 0.2	kap = 0.2	kap = 0.2	kap = 0.2	kap = 0.2	kap = 0.2	kap = 0.2	kap = 0.2	kap = 0.2	kap = 0.2	kap = 0.2	kap = 0.2	kap = 0.2	kap = 0.2	kap = 0.2	kap = 0.2	kap = 0.2	

0	0.02661	0.03056	0.03146	0.03664	0.04321	0.03055	0.03442	0.03254	0.03581	0.04339	0.02488	0.03441	0.03476	0.04252	0.04361	0.03038	0.03741	0.03624	0.05008	0.0529	0.03187	0.03332	0.04351	0.05795	0.06277	0.03168	0.04132	0.04922	0.07033	0.08052	0.03162	0.04416	0.0614	0.08919	0.10332	0.03911	0.04802	0.07774	0.10935	0.12951	0.04224	0.05134	0.0982	0.14548	0.17034	
40	0.02639	0.03007	0.03147	0.0367	0.04362	0.03057	0.03453	0.03252	0.03618	0.04361	0.02586	0.03439	0.03492	0.04252	0.0438	0.0301	0.03756	0.03623	0.04984	0.05284	0.03183	0.03304	0.04349	0.05797	0.06273	0.03183	0.04107	0.04951	0.07011	0.08027	0.03183	0.04411	0.06165	0.08904	0.1029	0.0396	0.04751	0.07767	0.10841	0.12895	0.04229	0.05115	0.09785	0.14457	0.16972	
80	0.0264	0.02977	0.03179	0.03723	0.04449	0.03054	0.03446	0.03271	0.03711	0.0443	0.02669	0.03454	0.03547	0.04312	0.04469	0.03029	0.03759	0.03673	0.05014	0.05338	0.03194	0.03307	0.04394	0.05883	0.06347	0.03198	0.0409	0.05039	0.07081	0.08091	0.03233	0.04448	0.06279	0.09009	0.10369	0.03992	0.0473	0.07875	0.10939	0.13006	0.04222	0.05143	0.09877	0.14585	0.17134	
120	0.02666	0.02983	0.03239	0.03823	0.04578	0.03051	0.03428	0.03317	0.03857	0.04547	0.0273	0.03485	0.03641	0.04437	0.04627	0.03099	0.03753	0.03777	0.05108	0.05459	0.03217	0.03347	0.04495	0.06055	0.06508	0.03221	0.04093	0.0519	0.0726	0.08262	0.03315	0.04527	0.06487	0.09251	0.10595	0.04008	0.04758	0.08109	0.11264	0.13317	0.04197	0.0523	0.10122	0.14975	0.17552	
160	0.02716	0.03041	0.0332	0.03967	0.0474	0.03058	0.03411	0.03395	0.04046	0.04709	0.02769	0.03533	0.03771	0.04626	0.0485	0.03215	0.0375	0.03933	0.05274	0.0565	0.03249	0.03428	0.04655	0.06312	0.06761	0.03263	0.04132	0.05406	0.07556	0.08552	0.03427	0.04644	0.06785	0.09636	0.10984	0.04013	0.04852	0.08469	0.11835	0.13848	0.04154	0.05383	0.10533	0.15643	0.18236	
200	0.02792	0.03155	0.03417	0.04149	0.04927	0.0308	0.03409	0.03508	0.04267	0.04909	0.02791	0.03597	0.03928	0.04867	0.05126	0.03365	0.03765	0.04136	0.05512	0.05911	0.03288	0.03553	0.04876	0.06643	0.07102	0.03331	0.04219	0.05688	0.0797	0.08966	0.03562	0.04795	0.07172	0.10162	0.11547	0.04018	0.05022	0.08949	0.12645	0.14607	0.04098	0.05599	0.1111	0.16585	0.19171	
240	0.02897	0.03316	0.03521	0.04359	0.05129	0.03123	0.03437	0.03655	0.04507	0.05138	0.02808	0.03673	0.04106	0.05143	0.05444	0.03533	0.03813	0.04375	0.05815	0.06238	0.03338	0.03721	0.05154	0.07035	0.07525	0.03431	0.04363	0.06033	0.08496	0.09502	0.03711	0.04979	0.07641	0.10824	0.12284	0.04031	0.05269	0.0954	0.1367	0.15584	0.04044	0.05874	0.11838	0.17769	0.20323	
280	0.03032	0.03506	0.0363	0.04589	0.05341	0.03192	0.03504	0.03833	0.04758	0.05387	0.02829	0.03757	0.04295	0.05437	0.05792	0.03698	0.03907	0.04643	0.06174	0.06622	0.03399	0.03931	0.05485	0.07475	0.08021	0.03562	0.04562	0.06437	0.09121	0.10156	0.03866	0.05192	0.08187	0.11612	0.13192	0.0406	0.05587	0.10231	0.14872	0.16759	0.04017	0.062	0.12699	0.19148	0.21644	
320	0.03194	0.03698	0.03745	0.04833	0.05559	0.03285	0.03613	0.04037	0.05015	0.05648	0.02864	0.03848	0.0449	0.05733	0.06158	0.03845	0.04053	0.04931	0.06575	0.07056	0.03476	0.04179	0.05864	0.07951	0.08581	0.03719	0.0481	0.06894	0.09832	0.1092	0.04016	0.05437	0.08805	0.12514	0.14256	0.0411	0.05962	0.11015	0.16203	0.18103	0.04045	0.06567	0.1367	0.20662	0.23076	
360	0.03379	0.0387	0.03874	0.05089	0.05785	0.034	0.03761	0.0426	0.05279	0.05917	0.02919	0.03945	0.04693	0.06023	0.06535	0.03963	0.04251	0.05236	0.07003	0.0753	0.03569	0.04461	0.06286	0.08457	0.09199	0.03891	0.05095	0.07396	0.10613	0.11785	0.04158	0.05717	0.0949	0.13522	0.15461	0.04183	0.06376	0.11883	0.17615	0.19579	0.04151	0.0697	0.14734	0.22246	0.24565	
400	0.03574	0.04002	0.0403	0.05356	0.06022	0.03531	0.0394	0.04495	0.05553	0.06193	0.02998	0.04053	0.04906	0.06308	0.06918	0.04049	0.04493	0.05555	0.07446	0.08039	0.03678	0.0477	0.06747	0.08991	0.0987	0.04067	0.054	0.0794	0.11455	0.12747	0.04288	0.06034	0.10237	0.14626	0.16787	0.04279	0.06813	0.1283	0.19066	0.21144	0.04352	0.07411	0.15877	0.2384	0.2606	
440	0.03762	0.04092	0.04228	0.05638	0.06275	0.03673	0.04138	0.04737	0.05844	0.06481	0.03103	0.04177	0.05138	0.06598	0.07308	0.04109	0.04766	0.0589	0.07897	0.08578	0.03802	0.05101	0.07241	0.09557	0.10597	0.04238	0.05717	0.08523	0.12351	0.13801	0.04411	0.06392	0.11046	0.15817	0.18209	0.044	0.0726	0.1385	0.20521	0.22757	0.04647	0.07897	0.17091	0.25389	0.2752	
480	0.03925	0.04147	0.0448	0.05938	0.0655	0.03819	0.04345	0.04985	0.06159	0.06786	0.03235	0.04325	0.05399	0.06907	0.07708	0.04154	0.05058	0.06246	0.08357	0.09147	0.0394	0.05448	0.07765	0.10166	0.11384	0.04401	0.06038	0.09146	0.13301	0.14944	0.04536	0.06794	0.11915	0.17084	0.19701	0.0455	0.07714	0.14932	0.21955	0.24374	0.05021	0.08441	0.18368	0.2685	0.2891	
520	0.04049	0.04188	0.04793	0.06262	0.06854	0.03963	0.04555	0.05242	0.06502	0.07118	0.03398	0.04505	0.057	0.07253	0.08126	0.04201	0.05359	0.06628	0.08831	0.09749	0.0409	0.05807	0.08316	0.10831	0.12239	0.04558	0.0637	0.09818	0.14306	0.16171	0.04672	0.0724	0.12847	0.18413	0.21236	0.04733	0.08181	0.16066	0.23353	0.25954	0.0544	0.09059	0.19698	0.28192	0.30204	
560	0.04124	0.04242	0.05164	0.06614	0.07192	0.04098	0.04767	0.05515	0.06877	0.07486	0.03594	0.04723	0.06048	0.07649	0.0857	0.04265	0.05663	0.07044	0.09334	0.10396	0.04256	0.06176	0.08892	0.11567	0.13172	0.04718	0.06722	0.10551	0.15371	0.17476	0.04833	0.07733	0.13846	0.19785	0.22784	0.04959	0.08676	0.17237	0.24703	0.27458	0.05863	0.09768	0.21062	0.29391	0.3138	
600	0.04155	0.04337	0.05585	0.06995	0.07568	0.04221	0.0499	0.05812	0.07288	0.07901	0.03825	0.04981	0.06451	0.08105	0.09055	0.04359	0.05974	0.075	0.09886	0.111	0.04442	0.06556	0.09494	0.12389	0.14192	0.04897	0.07112	0.11363	0.16499	0.18847	0.05031	0.08274	0.14914	0.21174	0.24313	0.05235	0.0922	0.18432	0.25996	0.28852	0.0625	0.10574	0.22433	0.30436	0.32417	
640	0.04155	0.04493	0.06036	0.07408	0.07987	0.04328	0.05234	0.06146	0.07736	0.08368	0.04093	0.05279	0.06909	0.08624	0.09595	0.0449	0.06299	0.08006	0.10506	0.11879	0.04655	0.06955	0.10129	0.1331	0.15305	0.05109	0.07555	0.12271	0.17691	0.20265	0.05275	0.08868	0.16056	0.22556	0.25792	0.0557	0.09837	0.19635	0.2722	0.30107	0.0657	0.11478	0.23769	0.31318	0.33298	
680	0.04149	0.0472	0.065	0.07855	0.08454	0.04424	0.05516	0.06526	0.08225	0.08896	0.04393	0.05613	0.07423	0.09204	0.10205	0.04659	0.06652	0.0857	0.11217	0.12751	0.04905	0.0738	0.10806	0.14339	0.16512	0.05368	0.08065	0.13289	0.18943	0.21707	0.05572	0.09518	0.17269	0.23902	0.27193	0.05965	0.10548	0.20836	0.28358	0.31202	0.0681	0.12467	0.25024	0.32034	0.34007	
720	0.04166	0.0502	0.0696	0.08337	0.08977	0.04513	0.0585	0.06963	0.08759	0.09488	0.04718	0.0598	0.07989	0.09847	0.10902	0.04861	0.07044	0.092	0.12036	0.13734	0.05199	0.07844	0.11541	0.15479	0.1781	0.05678	0.08647	0.14426	0.20247	0.23147	0.05923	0.10229	0.18546	0.25187	0.28487	0.0642	0.11367	0.22024	0.29391	0.32123	0.0698	0.13516	0.26151	0.32585	0.34531	
760	0.04235	0.05384	0.0741	0.0886	0.09562	0.04609	0.06244	0.07464	0.09347	0.1015	0.05058	0.06374	0.08604	0.10555	0.11703	0.0509	0.07487	0.09908	0.12976	0.14841	0.05544	0.08359	0.1235	0.16728	0.19191	0.06036	0.09298	0.15678	0.21589	0.24554	0.06324	0.11002	0.19869	0.26392	0.29653	0.06928	0.12296	0.23194	0.30296	0.32864	0.07108	0.1459	0.27109	0.32973	0.34865	
800	0.04379	0.05799	0.07853	0.0943	0.10221	0.04729	0.06704	0.08034	0.10001	0.10888	0.05402	0.06797	0.0927	0.11342	0.12624	0.05338	0.07986	0.10702	0.14045	0.16081	0.05944	0.08936	0.13253	0.18076	0.20639	0.06432	0.10011	0.17032	0.22952	0.25904	0.06769	0.11836	0.21216	0.27504	0.30676	0.0748	0.13328	0.24335	0.31052	0.33432	0.07238	0.15652	0.27875	0.33203	0.35013	
840	0.04609	0.06255	0.08307	0.10057	0.10963	0.04892	0.07226	0.08676	0.10735	0.11708	0.0574	0.07251	0.09988	0.12227	0.13677	0.05604	0.08543	0.11595	0.15246	0.17451	0.06401	0.09584	0.14266	0.19509	0.22134	0.06849	0.10776	0.1846	0.24315	0.27172	0.07244	0.12725	0.22555	0.28517	0.31549	0.08062	0.14442	0.25435	0.31643	0.33837	0.07417	0.16665	0.28445	0.33286	0.34988	
880	0.04921	0.06746	0.08798	0.10756	0.11801	0.05117	0.07803	0.09393	0.11563	0.12625	0.06066	0.07746	0.10768	0.13238	0.14873	0.05888	0.09155	0.12593	0.16577	0.18944	0.0691	0.10306	0.15401	0.21006	0.23653	0.07268	0.11585	0.19928	0.25659	0.28346	0.07735	0.13658	0.23852	0.29431	0.32272	0.08662	0.15606	0.26476	0.32062	0.34098	0.07684	0.17604	0.28835	0.33235	0.34812	
920	0.05303	0.07272	0.09359	0.11542	0.12751	0.05419	0.0843	0.10189	0.125	0.13653	0.06386	0.08296	0.11622	0.14402	0.16218	0.06198	0.09823	0.13703	0.18034	0.20541	0.07465	0.11105	0.16664	0.22544	0.2517	0.07676	0.12437	0.21394	0.26962	0.29417	0.08227	0.14619	0.25073	0.30248	0.32852	0.09267	0.16785	0.2743	0.32314	0.34236	0.08065	0.18456	0.2908	0.33067	0.34515	
960	0.05734	0.0784	0.10022	0.12436	0.13833	0.05804	0.091	0.11072	0.13562	0.14813	0.0671	0.08915	0.12567	0.15742	0.17715	0.06544	0.10549	0.14931	0.1961	0.22219	0.08059	0.1198	0.18051	0.24101	0.2666	0.08069	0.1334	0.22819	0.28205	0.30387	0.08705	0.15594	0.26192	0.30973	0.33301	0.09868	0.17943	0.28266	0.32416	0.34272	0.08566	0.19224	0.29226	0.32803	0.34128	
1000	0.06194	0.08462	0.10815	0.13463	0.15069	0.06266	0.09816	0.12056	0.14762	0.16132	0.0706	0.09621	0.13625	0.17267	0.19361	0.06938	0.11345	0.16277	0.21296	0.23949	0.08681	0.12927	0.19553	0.25651	0.28101	0.08452	0.14308	0.24166	0.29368	0.3126	0.09163	0.16568	0.27191	0.31607	0.33635	0.10457	0.19044	0.28949	0.32392	0.34226	0.09174	0.19926	0.29315	0.32466	0.33682	
1040	0.06668	0.09148	0.11761	0.14653	0.16489	0.06786	0.10591	0.13158	0.1612	0.17636	0.07458	0.10432	0.14818	0.18972	0.21152	0.07392	0.12228	0.17741	0.23077	0.25704	0.09322	0.13948	0.21154	0.27175	0.29473	0.08843	0.15358	0.25413	0.30435	0.32044	0.09599	0.17534	0.28063	0.32146	0.3387	0.11028	0.20064	0.29454	0.32273	0.34112	0.09861	0.20591	0.29386	0.32076	0.33203	
1080	0.07148	0.09911	0.12877	0.1604	0.18126	0.07337	0.11443	0.14404	0.17654	0.19352	0.07928	0.11365	0.16169	0.20834	0.23078	0.07912	0.13225	0.19322	0.24936	0.27456	0.09971	0.15043	0.2283	0.28651	0.30763	0.09266	0.16504	0.26547	0.31392	0.32749	0.10022	0.18492	0.28812	0.32585	0.34019	0.11578	0.20988	0.29763	0.32092	0.33941	0.10596	0.21251	0.29457	0.31653	0.32708	
1120	0.07638	0.10763	0.14177	0.17661	0.20013	0.07887	0.12401	0.15823	0.19389	0.21305	0.08486	0.12436	0.17697	0.22817	0.25127	0.08501	0.14363	0.21015	0.26848	0.29184	0.10621	0.16218	0.24559	0.30063	0.31959	0.09747	0.17754	0.2757	0.32227	0.33379	0.1045	0.19447	0.29451	0.32915	0.34095	0.12107	0.21813	0.29879	0.31874	0.33722	0.11352	0.21933	0.29529	0.31214	0.32209	
1160	0.08148	0.11718	0.1568	0.19555	0.22185	0.08406	0.13497	0.17448	0.21351	0.23511	0.0914	0.13664	0.19418	0.24879	0.27285	0.09156	0.15667	0.22816	0.2878	0.3087	0.11265	0.17481	0.26312	0.31395	0.33059	0.10309	0.191	0.28493	0.32931	0.33938	0.1091	0.20412	0.29996	0.33131	0.34105	0.12615	0.22548	0.29826	0.3164	0.33456	0.12113	0.22654	0.29585	0.30769	0.3171	
1200	0.08689	0.12799	0.17414	0.2176	0.24665	0.08876	0.14762	0.19314	0.23565	0.25976	0.09882	0.15067	0.21342	0.26981	0.29534	0.09872	0.17154	0.24717	0.30698	0.32502	0.11898	0.18838	0.28063	0.32632	0.34061	0.10967	0.20524	0.29338	0.33502	0.34422	0.11432	0.21402	0.30463	0.33228	0.34057	0.13109	0.23212	0.29645	0.31399	0.33148	0.12876	0.23414	0.29599	0.30327	0.31211	
1240	0.09268	0.1404	0.19413	0.24301	0.27461	0.09292	0.16223	0.21451	0.2605	0.28689	0.10696	0.16667	0.23469	0.29094	0.31851	0.10642	0.18828	0.26706	0.32561	0.3407	0.12521	0.20295	0.29782	0.33757	0.34967	0.11722	0.21998	0.30126	0.33942	0.34828	0.12048	0.22426	0.30862	0.33214	0.33953	0.13599	0.23832	0.2939	0.31152	0.32802	0.1365	0.24194	0.2954	0.2989	0.30712	
1280	0.09885	0.15487	0.21718	0.2719	0.30558	0.09671	0.17898	0.23882	0.28809	0.31621	0.11552	0.18481	0.25791	0.31205	0.3421	0.11458	0.20678	0.28765	0.34332	0.3557	0.13137	0.21851	0.3144	0.34757	0.3578	0.12564	0.23486	0.30875	0.34259	0.3515	0.12786	0.23489	0.31196	0.33101	0.33797	0.141	0.24438	0.29122	0.30892	0.32424	0.14451	0.24962	0.29386	0.29458	0.30213	
1320	0.10527	0.17191	0.24366	0.30412	0.33911	0.10043	0.19794	0.26614	0.31823	0.34721	0.1242	0.20523	0.28284	0.33313	0.36575	0.12317	0.22678	0.30868	0.35979	0.36992	0.13754	0.23499	0.33006	0.35618	0.36504	0.13474	0.24955	0.31599	0.34469	0.35387	0.13664	0.24584	0.31461	0.32912	0.33595	0.1463	0.25054	0.28892	0.30612	0.32021	0.1529	0.25679	0.29127	0.29029	0.29717	
1360	0.11171	0.19204	0.27375	0.33921	0.37445	0.10452	0.21905	0.29634	0.35042	0.37916	0.13265	0.22794	0.30913	0.35426	0.38905	0.13215	0.24788	0.32979	0.37478	0.3833	0.14383	0.25219	0.34451	0.36331	0.37143	0.14426	0.26379	0.323	0.34593	0.35537	0.14685	0.25693	0.31653	0.32676	0.33355	0.1521	0.25701	0.2874	0.30307	0.31604	0.1617	0.26306	0.28772	0.28603	0.29232	
1400	0.11789	0.21559	0.30732	0.37635	0.4105	0.10941	0.24209	0.32902	0.38386	0.41114	0.14063	0.25274	0.33628	0.37553	0.41152	0.14148	0.2696	0.35052	0.3882	0.3957	0.15034	0.26986	0.35746	0.36895	0.377	0.15395	0.27746	0.32973	0.34653	0.35607	0.15837	0.26789	0.31764	0.32424	0.33088	0.15856	0.26389	0.2868	0.29975	0.31185	0.17082	0.26811	0.2835	0.28179	0.28765	
1440	0.12355	0.24255	0.34377	0.41438	0.44595	0.1154	0.26666	0.36348	0.41742	0.44206	0.14795	0.27921	0.36367	0.39689	0.43262	0.15109	0.29141	0.37037	0.40002	0.40697	0.15713	0.28763	0.36871	0.37315	0.38178	0.16359	0.29053	0.33606	0.34676	0.35608	0.17094	0.27839	0.31795	0.32185	0.32808	0.16575	0.27113	0.28706	0.29626	0.30774	0.18001	0.27181	0.27898	0.2776	0.28326	
1480	0.12849	0.27248	0.382	0.45182	0.47932	0.12262	0.29221	0.3987	0.44974	0.47077	0.15453	0.30659	0.39054	0.41807	0.45176	0.16082	0.31276	0.38876	0.41031	0.41695	0.16421	0.30507	0.37813	0.3761	0.38579	0.17303	0.30305	0.34181	0.34683	0.35555	0.18411	0.28813	0.31753	0.31982	0.32526	0.17364	0.27857	0.28794	0.29276	0.3038	0.18894	0.27421	0.27462	0.27353	0.2792	
1520	0.13264	0.30436	0.42037	0.48699	0.50908	0.13091	0.31799	0.43334	0.47932	0.4961	0.16036	0.33387	0.41608	0.43854	0.46837	0.17043	0.33307	0.40513	0.41915	0.42543	0.17147	0.32173	0.38567	0.37803	0.38907	0.18216	0.3151	0.34681	0.34693	0.35467	0.19736	0.29681	0.31656	0.31829	0.32258	0.18204	0.28594	0.28907	0.28946	0.30012	0.19727	0.27555	0.27084	0.26965	0.27549	
1560	0.13602	0.33661	0.45684	0.51814	0.53384	0.13983	0.34308	0.46585	0.50469	0.51696	0.16548	0.35979	0.43941	0.45749	0.48189	0.17958	0.35179	0.41896	0.42661	0.43223	0.17871	0.33713	0.39143	0.37927	0.39164	0.19092	0.32668	0.35092	0.34717	0.35362	0.21011	0.30427	0.31531	0.31728	0.32012	0.19064	0.29291	0.29009	0.2866	0.29673	0.20476	0.2762	0.26797	0.26605	0.27215	
1600	0.13879	0.36724	0.48916	0.54361	0.55245	0.14873	0.3664	0.49457	0.52456	0.53245	0.1699	0.38297	0.45965	0.4739	0.49185	0.18783	0.36831	0.4298	0.43263	0.43716	0.1856	0.35078	0.39556	0.38012	0.39349	0.19918	0.33766	0.35403	0.34757	0.35254	0.22174	0.31042	0.31406	0.31672	0.31794	0.19897	0.29912	0.29071	0.28437	0.29366	0.21127	0.27659	0.26621	0.26285	0.26912	
1640	0.1411	0.39405	0.51514	0.562	0.5641	0.15681	0.38679	0.51784	0.53797	0.54193	0.17362	0.40202	0.47597	0.48672	0.49789	0.19471	0.38204	0.43736	0.43705	0.44006	0.19176	0.36224	0.3983	0.38086	0.39463	0.2068	0.34773	0.35612	0.34807	0.35152	0.23167	0.31528	0.31309	0.31644	0.31603	0.20655	0.30426	0.29083	0.28289	0.29091	0.21683	0.27712	0.26558	0.26014	0.26636	
1680	0.14308	0.41487	0.53295	0.5723	0.5684	0.16331	0.40306	0.53423	0.54436	0.54507	0.17657	0.41574	0.48768	0.49497	0.49977	0.19976	0.39234	0.44144	0.43961	0.4408	0.19677	0.37108	0.39985	0.3816	0.39501	0.21349	0.35639	0.35721	0.34854	0.35059	0.23941	0.3189	0.31257	0.31624	0.31432	0.21289	0.3081	0.29047	0.28217	0.28848	0.22158	0.27805	0.266	0.25798	0.26381	
1720	0.14475	0.42793	0.54132	0.57403	0.56541	0.16759	0.41413	0.54266	0.54368	0.54188	0.1786	0.42324	0.49421	0.4979	0.49742	0.2026	0.39858	0.44201	0.43993	0.43926	0.20027	0.37693	0.40034	0.38232	0.39452	0.21891	0.36305	0.35737	0.34876	0.34966	0.24454	0.32135	0.31254	0.31593	0.31269	0.2176	0.31051	0.28983	0.28209	0.28636	0.22572	0.27945	0.26726	0.25641	0.26145	
1760	0.14598	0.43208	0.53977	0.56722	0.55555	0.16929	0.41911	0.54256	0.53629	0.53268	0.17952	0.42404	0.49519	0.49514	0.49092	0.20303	0.40025	0.43912	0.43764	0.43536	0.202	0.37947	0.39978	0.38278	0.39302	0.22264	0.36707	0.35665	0.34851	0.34857	0.24676	0.32266	0.31289	0.31532	0.311	0.22042	0.31149	0.28918	0.28243	0.28452	0.22935	0.28117	0.26913	0.2554	0.25927	
1800	0.14649	0.42696	0.52863	0.55244	0.5396	0.16834	0.41746	0.53392	0.52293	0.51807	0.17915	0.41815	0.49048	0.48675	0.48049	0.20103	0.39696	0.43291	0.43243	0.42907	0.20188	0.37846	0.39802	0.38259	0.39033	0.22427	0.3679	0.3551	0.34751	0.34708	0.24593	0.32276	0.31336	0.31426	0.30908	0.22127	0.31113	0.28873	0.28291	0.28292	0.23245	0.28289	0.27136	0.25489	0.2573	
1840	0.14593	0.41307	0.50897	0.53074	0.51853	0.165	0.40908	0.51736	0.5046	0.49888	0.17732	0.40605	0.48019	0.47318	0.46648	0.19677	0.38859	0.4236	0.42414	0.42038	0.19996	0.37377	0.39475	0.38121	0.38623	0.22345	0.36515	0.35271	0.34551	0.3449	0.24202	0.32151	0.31357	0.31266	0.30675	0.22023	0.30956	0.28862	0.2832	0.28149	0.23474	0.28412	0.27368	0.25477	0.25555	
1880	0.14395	0.39168	0.48246	0.50345	0.49344	0.15975	0.39432	0.49398	0.48237	0.47602	0.17396	0.38864	0.46466	0.45519	0.44933	0.19063	0.37532	0.41141	0.41281	0.40935	0.19647	0.36542	0.38959	0.37809	0.3805	0.22002	0.35868	0.3494	0.34227	0.34173	0.2352	0.31872	0.3131	0.31048	0.30387	0.21751	0.30694	0.28879	0.283	0.28013	0.23577	0.28434	0.27587	0.25488	0.25405	
1920	0.1403	0.36464	0.45109	0.47213	0.46545	0.15321	0.37402	0.46532	0.45734	0.45047	0.16907	0.36708	0.44447	0.43378	0.42955	0.18311	0.3577	0.3966	0.39871	0.39609	0.19172	0.3536	0.38208	0.37271	0.37299	0.214	0.34863	0.34501	0.33758	0.33729	0.22577	0.31415	0.31155	0.30765	0.30031	0.21335	0.30335	0.28899	0.28208	0.2787	0.23492	0.28308	0.2777	0.25503	0.25278	
1960	0.13489	0.3341	0.41695	0.43833	0.43561	0.14601	0.34937	0.43312	0.43044	0.42317	0.1628	0.34267	0.42038	0.40998	0.40769	0.17476	0.33659	0.37947	0.38229	0.3808	0.18605	0.33864	0.37188	0.3647	0.36356	0.20565	0.33539	0.33932	0.33132	0.33133	0.2142	0.30761	0.30861	0.30416	0.29599	0.20803	0.29879	0.28883	0.28028	0.27703	0.23159	0.27996	0.27892	0.25502	0.25166	
2000	0.12787	0.30222	0.3819	0.40353	0.40484	0.13868	0.32187	0.39913	0.40246	0.39494	0.15538	0.31669	0.39333	0.38475	0.38431	0.16611	0.31311	0.36032	0.36415	0.36373	0.17974	0.32107	0.35877	0.3539	0.3522	0.19541	0.31951	0.33209	0.32338	0.32371	0.2011	0.29901	0.30406	0.29991	0.29082	0.20175	0.29316	0.28778	0.27753	0.2749	0.2253	0.27482	0.27926	0.25464	0.25057	
2040	0.11962	0.27091	0.34747	0.36896	0.37392	0.13154	0.29304	0.36496	0.37399	0.36648	0.1471	0.29029	0.36432	0.35891	0.35994	0.15759	0.28851	0.33954	0.3449	0.34522	0.17299	0.30155	0.34277	0.34041	0.33901	0.18387	0.30168	0.32308	0.31376	0.31439	0.18713	0.28835	0.29783	0.29482	0.28478	0.19461	0.28628	0.28537	0.27381	0.27209	0.21585	0.26767	0.27843	0.25371	0.24932	
2080	0.11067	0.24165	0.31473	0.33562	0.3435	0.12475	0.26436	0.33187	0.34547	0.33834	0.1383	0.2644	0.3344	0.33308	0.33512	0.14947	0.26402	0.31753	0.32515	0.32564	0.16585	0.28082	0.32418	0.32455	0.32419	0.17166	0.28259	0.31211	0.30252	0.30342	0.17302	0.27583	0.28996	0.28875	0.27783	0.18668	0.27792	0.2812	0.26915	0.26841	0.20342	0.25871	0.27615	0.25204	0.2477	
2120	0.10159	0.21538	0.28428	0.30417	0.31405	0.11826	0.23706	0.30076	0.31722	0.31093	0.12931	0.2397	0.30455	0.30769	0.31031	0.14187	0.2407	0.29479	0.30534	0.30542	0.15825	0.25964	0.30352	0.30683	0.30802	0.15936	0.26287	0.29909	0.28979	0.29097	0.15941	0.26175	0.28055	0.28158	0.26996	0.17796	0.2679	0.27503	0.26359	0.26367	0.18859	0.24828	0.27217	0.24947	0.24548	
2160	0.09296	0.19252	0.25637	0.27501	0.28597	0.11192	0.21202	0.27209	0.28954	0.28457	0.12042	0.21661	0.27566	0.28302	0.28594	0.13474	0.21932	0.2718	0.28577	0.28495	0.15006	0.2387	0.28151	0.28788	0.29088	0.14742	0.24305	0.28409	0.27579	0.2773	0.14684	0.24651	0.26979	0.27317	0.26117	0.16848	0.25609	0.26684	0.25714	0.25774	0.17223	0.23679	0.26631	0.24587	0.24242	
2200	0.08517	0.17308	0.23095	0.24831	0.25951	0.10557	0.18973	0.24598	0.26269	0.25946	0.11183	0.19539	0.24843	0.25926	0.26236	0.12795	0.20029	0.24911	0.26656	0.26462	0.14116	0.21859	0.25896	0.26837	0.27314	0.13614	0.22354	0.26732	0.26076	0.26273	0.13564	0.23058	0.25784	0.26344	0.25148	0.15831	0.24253	0.2568	0.24979	0.25056	0.15543	0.22466	0.2585	0.24115	0.23835	
2240	0.07845	0.15673	0.20787	0.22405	0.23487	0.09909	0.17031	0.22229	0.23699	0.23577	0.10371	0.17611	0.22339	0.23659	0.23989	0.12128	0.18366	0.22718	0.24772	0.24476	0.13151	0.19972	0.23667	0.24887	0.2552	0.12563	0.20465	0.24916	0.24503	0.24759	0.12594	0.21439	0.24489	0.25238	0.24093	0.14766	0.22742	0.24521	0.2415	0.24216	0.13927	0.21227	0.24882	0.23525	0.23314	
2280	0.0728	0.14299	0.18695	0.20212	0.21217	0.09249	0.15362	0.20074	0.21275	0.21364	0.09614	0.15881	0.20081	0.21518	0.21874	0.11455	0.16917	0.20645	0.22917	0.22564	0.12122	0.18234	0.21533	0.22988	0.23739	0.11583	0.18661	0.23012	0.22894	0.23222	0.11764	0.19836	0.23112	0.24006	0.22962	0.13683	0.21114	0.23247	0.23222	0.23261	0.12467	0.19991	0.23749	0.22816	0.22673	
2320	0.06806	0.13133	0.16807	0.18233	0.19144	0.0859	0.13932	0.18102	0.19028	0.19315	0.08912	0.14344	0.18078	0.1952	0.19908	0.10761	0.15637	0.18721	0.21088	0.20745	0.11055	0.1665	0.19547	0.21171	0.22	0.10663	0.1696	0.21081	0.21281	0.21691	0.11046	0.18278	0.21673	0.22663	0.21765	0.12619	0.19421	0.21898	0.22196	0.22206	0.11224	0.18777	0.22483	0.21992	0.21913	
2360	0.06399	0.12125	0.15118	0.16449	0.17268	0.07952	0.12698	0.16292	0.16986	0.17439	0.08265	0.12996	0.16323	0.17682	0.18101	0.10039	0.14475	0.16966	0.19289	0.19032	0.09992	0.15215	0.17739	0.19455	0.20324	0.09787	0.15377	0.19188	0.19696	0.2019	0.10402	0.16789	0.20194	0.21236	0.20519	0.11614	0.17721	0.2051	0.21077	0.21072	0.10221	0.17598	0.21122	0.21062	0.21043	
2400	0.06035	0.11235	0.13627	0.14844	0.15584	0.07364	0.11624	0.1463	0.15166	0.1574	0.07668	0.11832	0.14793	0.16018	0.16456	0.09295	0.13384	0.15384	0.17536	0.17429	0.08982	0.13915	0.16118	0.17845	0.18728	0.08944	0.13926	0.17393	0.18168	0.18738	0.09792	0.15383	0.18698	0.19754	0.19244	0.10703	0.16072	0.19113	0.19879	0.19883	0.09445	0.16457	0.1971	0.20041	0.2008	
2440	0.05698	0.10431	0.12335	0.13405	0.1408	0.06846	0.10679	0.13115	0.13575	0.14217	0.07116	0.10842	0.13459	0.14533	0.14969	0.08541	0.12334	0.1397	0.15855	0.15939	0.08071	0.12731	0.14678	0.16342	0.17218	0.08134	0.12615	0.15746	0.16715	0.17346	0.09183	0.14067	0.17216	0.18253	0.17963	0.0991	0.14525	0.17727	0.18626	0.18662	0.08853	0.15357	0.18286	0.1895	0.19044	
2480	0.05383	0.09687	0.11237	0.12122	0.12744	0.06414	0.09843	0.11755	0.12208	0.12865	0.06603	0.10013	0.12288	0.13223	0.13636	0.07793	0.11312	0.1271	0.14282	0.14562	0.07298	0.11649	0.13402	0.14941	0.15802	0.07362	0.11448	0.14279	0.15354	0.1602	0.08557	0.12845	0.15776	0.16767	0.16697	0.09242	0.13117	0.16368	0.17347	0.17435	0.08386	0.14298	0.16883	0.17813	0.1796	
2520	0.05099	0.08988	0.10319	0.10984	0.11562	0.06067	0.09107	0.10558	0.11048	0.11672	0.06128	0.09326	0.11252	0.12075	0.12444	0.07073	0.10326	0.11583	0.12851	0.13295	0.06678	0.10658	0.12267	0.13638	0.14482	0.06645	0.10419	0.13008	0.1409	0.14766	0.07916	0.11719	0.14408	0.1533	0.15469	0.08689	0.11868	0.15049	0.16076	0.16225	0.07983	0.13282	0.15525	0.16655	0.16853	
2560	0.04861	0.08319	0.09556	0.09985	0.10517	0.05793	0.08463	0.09528	0.1007	0.10624	0.05689	0.08757	0.10323	0.11067	0.11382	0.064	0.09397	0.10571	0.11589	0.12134	0.06206	0.09753	0.11252	0.12433	0.13257	0.06	0.09519	0.11928	0.12926	0.13584	0.07277	0.1069	0.13133	0.1397	0.14295	0.08228	0.1078	0.13781	0.14845	0.15053	0.07593	0.12311	0.1423	0.15503	0.15749	
2600	0.04688	0.07676	0.08917	0.09114	0.09594	0.05568	0.07904	0.08662	0.09243	0.09701	0.05288	0.08277	0.09482	0.10175	0.10436	0.05789	0.08548	0.09653	0.10508	0.11076	0.05855	0.0893	0.10338	0.11328	0.12127	0.05443	0.08728	0.11015	0.11859	0.12478	0.06669	0.09756	0.11966	0.12706	0.13187	0.07825	0.09843	0.12579	0.13682	0.13933	0.07185	0.11388	0.13009	0.14379	0.14672	
2640	0.04591	0.07059	0.08366	0.08361	0.08781	0.05364	0.07417	0.07945	0.08538	0.08885	0.04927	0.07858	0.08712	0.09375	0.0959	0.05252	0.07804	0.08815	0.09606	0.10115	0.05584	0.0819	0.09512	0.10324	0.1109	0.04984	0.08028	0.10236	0.10882	0.11447	0.06125	0.08915	0.1091	0.11551	0.12154	0.07447	0.09034	0.11458	0.12601	0.1288	0.06748	0.10518	0.11867	0.133	0.1364	
2680	0.04569	0.06474	0.0787	0.07712	0.08064	0.05155	0.06985	0.07352	0.07924	0.08157	0.04611	0.07472	0.08002	0.08648	0.08833	0.04795	0.07176	0.08047	0.08859	0.09245	0.05345	0.0753	0.08766	0.09424	0.10143	0.0462	0.074	0.09551	0.09989	0.10496	0.05673	0.08163	0.09959	0.10511	0.11195	0.07066	0.0833	0.1043	0.1161	0.11898	0.06289	0.09703	0.10809	0.12277	0.12666	
2720	0.04609	0.05934	0.07401	0.0715	0.07431	0.04918	0.06587	0.06853	0.07381	0.07501	0.04344	0.07097	0.07344	0.07979	0.08151	0.0442	0.06663	0.07343	0.08235	0.08462	0.05096	0.06947	0.08091	0.08624	0.09279	0.04342	0.06828	0.08921	0.09172	0.09624	0.05327	0.07491	0.091	0.09582	0.10308	0.06667	0.07706	0.09504	0.10701	0.10991	0.05828	0.08942	0.09838	0.11317	0.11754	
2760	0.04686	0.05454	0.0694	0.06658	0.06872	0.04646	0.06201	0.06419	0.0689	0.06906	0.04129	0.06718	0.06731	0.07359	0.07535	0.04125	0.0625	0.06699	0.07692	0.07756	0.04808	0.0643	0.07479	0.07917	0.08492	0.04129	0.06307	0.08316	0.08425	0.08833	0.05082	0.0689	0.08316	0.08757	0.09487	0.06246	0.07145	0.08681	0.09862	0.10156	0.05386	0.08235	0.08956	0.10418	0.10906	
2800	0.04764	0.05047	0.0648	0.0622	0.06377	0.04337	0.0581	0.06022	0.06441	0.06365	0.03964	0.06327	0.06158	0.06785	0.06975	0.03905	0.05913	0.06115	0.07192	0.07123	0.04473	0.05969	0.06922	0.07293	0.07775	0.03957	0.05837	0.07718	0.07743	0.08119	0.04916	0.06348	0.07593	0.08025	0.08725	0.05812	0.06634	0.07957	0.09076	0.09389	0.04982	0.07578	0.08165	0.09577	0.10114	
2840	0.04812	0.04719	0.06021	0.0582	0.05936	0.04003	0.05405	0.05645	0.06027	0.05875	0.03842	0.05924	0.05621	0.06253	0.06464	0.03755	0.05622	0.05589	0.06705	0.06554	0.04104	0.0555	0.0641	0.06737	0.07119	0.03804	0.05422	0.07122	0.07122	0.07477	0.04792	0.05855	0.06922	0.07375	0.08016	0.0538	0.06168	0.07318	0.08328	0.08683	0.04627	0.0697	0.07467	0.08791	0.0937	
2880	0.04802	0.04466	0.05571	0.05448	0.05541	0.03659	0.04987	0.05279	0.05643	0.05436	0.03751	0.05513	0.05118	0.05763	0.05998	0.03666	0.05349	0.05122	0.06214	0.06045	0.03725	0.05163	0.05932	0.06236	0.06518	0.03651	0.05069	0.06534	0.0656	0.06901	0.04666	0.05404	0.06303	0.06796	0.07357	0.0497	0.05746	0.06747	0.0761	0.08031	0.04323	0.06409	0.06857	0.08054	0.08666	
2920	0.04721	0.04275	0.05138	0.05095	0.05184	0.03322	0.04569	0.04924	0.05289	0.05049	0.03676	0.051	0.04651	0.0531	0.05572	0.03628	0.05071	0.04713	0.05719	0.05592	0.03371	0.04802	0.05483	0.05778	0.05968	0.03487	0.0478	0.05971	0.06055	0.06381	0.04499	0.0499	0.05742	0.06278	0.06746	0.04594	0.05368	0.06227	0.0692	0.07427	0.04066	0.05895	0.0633	0.07369	0.07995	
2960	0.04568	0.0412	0.04733	0.0476	0.0486	0.03007	0.0417	0.04587	0.04961	0.04713	0.03599	0.04693	0.04226	0.0489	0.05182	0.03628	0.04778	0.04359	0.05231	0.05191	0.03074	0.04463	0.05058	0.05355	0.05467	0.03311	0.04551	0.05451	0.05605	0.05908	0.04263	0.04611	0.05249	0.05814	0.06184	0.04261	0.05035	0.05743	0.06268	0.06867	0.03846	0.0543	0.05876	0.06738	0.07355	
3000	0.04352	0.03973	0.04363	0.04443	0.04562	0.02725	0.03813	0.04279	0.04658	0.04428	0.03502	0.04298	0.03848	0.04498	0.04825	0.03651	0.04467	0.04058	0.04771	0.04841	0.02852	0.04149	0.04659	0.04964	0.05015	0.0313	0.0437	0.04992	0.05206	0.05474	0.03949	0.04269	0.04836	0.05399	0.05671	0.0397	0.04744	0.05288	0.05669	0.06349	0.03654	0.05016	0.05485	0.06165	0.06748	
3040	0.04093	0.03806	0.0403	0.04148	0.04286	0.02481	0.03517	0.04005	0.04379	0.04189	0.03374	0.0392	0.03527	0.04132	0.04498	0.03679	0.04144	0.03808	0.04361	0.04537	0.0271	0.03866	0.04291	0.04605	0.04614	0.02955	0.04216	0.04605	0.04855	0.05073	0.03568	0.03966	0.04509	0.0503	0.05211	0.03714	0.04488	0.04861	0.05139	0.05872	0.03486	0.04655	0.05144	0.05657	0.0618	
3080	0.03808	0.03601	0.03734	0.03878	0.04029	0.02277	0.03293	0.03771	0.04118	0.03988	0.03211	0.03562	0.03271	0.03794	0.04196	0.03695	0.03822	0.03602	0.04015	0.04276	0.02634	0.0362	0.03962	0.04279	0.04266	0.02801	0.04068	0.04292	0.04548	0.04702	0.03149	0.03701	0.04267	0.04703	0.04802	0.03484	0.04261	0.04466	0.04693	0.05436	0.03337	0.04348	0.04841	0.05217	0.0566	
3120	0.03515	0.03351	0.03476	0.03637	0.03788	0.02113	0.03143	0.03572	0.03876	0.03816	0.03018	0.03226	0.03083	0.03488	0.03915	0.03682	0.03514	0.03437	0.03744	0.0405	0.02596	0.03414	0.03679	0.03988	0.03969	0.02678	0.03902	0.04047	0.04277	0.04359	0.02732	0.03472	0.04098	0.04414	0.04444	0.03272	0.04054	0.04113	0.04336	0.05041	0.03207	0.0409	0.04568	0.04842	0.05193	
3160	0.0322	0.03067	0.03251	0.03426	0.03558	0.01989	0.03058	0.03402	0.03648	0.03661	0.0281	0.02915	0.02961	0.03224	0.03652	0.03625	0.03231	0.03303	0.03542	0.0385	0.02562	0.03248	0.03443	0.0373	0.03721	0.02595	0.03704	0.03856	0.04038	0.04046	0.02359	0.03273	0.03981	0.04157	0.0413	0.03074	0.03856	0.03808	0.04065	0.04685	0.03095	0.03875	0.04318	0.04526	0.04785	
3200	0.02925	0.0277	0.03058	0.03241	0.03339	0.01905	0.03018	0.03249	0.03433	0.03514	0.02611	0.02631	0.02898	0.03009	0.03403	0.03515	0.02982	0.03193	0.03397	0.03668	0.02501	0.03117	0.03254	0.03501	0.03515	0.02551	0.03468	0.03701	0.03821	0.03764	0.02066	0.03092	0.03892	0.03926	0.03855	0.02892	0.03663	0.03555	0.03867	0.04366	0.02998	0.0369	0.04084	0.04255	0.04432	
3240	0.02626	0.0249	0.02895	0.03077	0.03127	0.0186	0.02999	0.03101	0.0323	0.03365	0.02445	0.02376	0.02878	0.0285	0.03168	0.03349	0.0277	0.03095	0.03289	0.03493	0.02391	0.0301	0.03101	0.03295	0.03342	0.0254	0.03199	0.03566	0.03621	0.03514	0.01875	0.02921	0.03805	0.03714	0.03611	0.02734	0.03469	0.03353	0.03717	0.04077	0.02909	0.03525	0.03864	0.04014	0.04128	
3280	0.02321	0.02255	0.0276	0.02929	0.02923	0.01854	0.02978	0.02948	0.03038	0.03209	0.02333	0.02153	0.02882	0.02749	0.02947	0.03131	0.02593	0.02999	0.03196	0.03319	0.02225	0.02914	0.02972	0.03104	0.03191	0.02552	0.02914	0.03435	0.03431	0.03295	0.01791	0.02751	0.037	0.03513	0.0339	0.02608	0.03273	0.03194	0.03591	0.03813	0.02819	0.03365	0.03652	0.03786	0.03862	
3320	0.02008	0.02087	0.02649	0.02789	0.02726	0.01883	0.02933	0.02783	0.02858	0.03044	0.02288	0.01962	0.02889	0.02698	0.02742	0.02872	0.02446	0.02897	0.03099	0.03139	0.02012	0.02817	0.02852	0.02921	0.03051	0.02571	0.02633	0.03299	0.03246	0.03105	0.01801	0.02577	0.03564	0.03321	0.03187	0.02521	0.03076	0.03064	0.03463	0.03567	0.02717	0.03199	0.03446	0.03558	0.03622	
3360	0.01695	0.01992	0.02558	0.0265	0.0254	0.0194	0.02851	0.02606	0.02691	0.02871	0.02311	0.01803	0.02878	0.02685	0.02557	0.02587	0.02322	0.02784	0.02986	0.02954	0.01774	0.02711	0.02726	0.02742	0.02915	0.02581	0.02376	0.03155	0.03064	0.0294	0.01879	0.02402	0.03397	0.03134	0.02999	0.02472	0.02878	0.02953	0.03316	0.03337	0.02596	0.03022	0.03242	0.03323	0.03397	
3400	0.01395	0.01963	0.02483	0.0251	0.02369	0.02014	0.02725	0.02421	0.02538	0.02697	0.02388	0.01674	0.02835	0.02691	0.02397	0.02298	0.02212	0.02657	0.02851	0.02767	0.01538	0.02592	0.02585	0.02569	0.02776	0.02569	0.0216	0.03006	0.02885	0.02798	0.01992	0.02232	0.03208	0.02955	0.02825	0.02452	0.02683	0.02848	0.03143	0.03121	0.02454	0.02832	0.0304	0.03083	0.03182	
3440	0.01127	0.01979	0.02419	0.0237	0.02218	0.02092	0.0256	0.02234	0.02401	0.02527	0.02492	0.0157	0.02751	0.02697	0.02264	0.02025	0.02109	0.02519	0.027	0.02584	0.01335	0.0246	0.02427	0.02404	0.02634	0.02522	0.01992	0.02857	0.0271	0.02672	0.02107	0.0208	0.03011	0.02789	0.02668	0.02444	0.02492	0.02743	0.02948	0.02921	0.02294	0.02633	0.0284	0.02848	0.02977	
3480	0.00912	0.02012	0.02359	0.02232	0.02091	0.02156	0.02362	0.02055	0.02281	0.02369	0.0259	0.01485	0.02626	0.02686	0.02163	0.01787	0.02005	0.02376	0.02541	0.02415	0.01183	0.02325	0.02257	0.02256	0.02491	0.02437	0.01868	0.02715	0.02544	0.02561	0.02197	0.01959	0.02824	0.02641	0.02531	0.02428	0.02309	0.02638	0.02743	0.0274	0.02128	0.02434	0.02645	0.02634	0.02788	
3520	0.00765	0.02036	0.02299	0.02101	0.01992	0.02196	0.02145	0.01892	0.0218	0.0223	0.02648	0.01415	0.02468	0.02645	0.0209	0.01601	0.01895	0.02235	0.02388	0.02266	0.01092	0.02195	0.02085	0.02131	0.02352	0.02312	0.01779	0.02583	0.0239	0.02461	0.02246	0.01878	0.0266	0.02517	0.02416	0.02381	0.02136	0.02536	0.02548	0.02582	0.0197	0.02247	0.02462	0.02458	0.02624	
3560	0.00692	0.0203	0.02237	0.01983	0.01918	0.02201	0.01924	0.01751	0.02094	0.02112	0.02637	0.01354	0.02289	0.02571	0.02042	0.01473	0.01778	0.02105	0.02251	0.02143	0.01059	0.02081	0.01925	0.02034	0.02222	0.02154	0.01708	0.02463	0.02252	0.02371	0.0225	0.01842	0.02526	0.0242	0.02324	0.02286	0.01977	0.0244	0.0238	0.02451	0.01835	0.02082	0.02297	0.02333	0.02492	
3600	0.0069	0.01985	0.02172	0.01881	0.01867	0.0217	0.01711	0.01634	0.02021	0.02017	0.02541	0.01299	0.02101	0.02469	0.02009	0.01406	0.01651	0.01992	0.02138	0.02046	0.01067	0.01991	0.01787	0.01966	0.02104	0.01974	0.01641	0.02353	0.02133	0.0229	0.02214	0.01848	0.0242	0.02347	0.0225	0.02136	0.01836	0.02353	0.02251	0.02344	0.01734	0.01949	0.02158	0.02264	0.02395	
3640	0.00742	0.01907	0.02103	0.01797	0.0183	0.02109	0.0152	0.01542	0.01955	0.01941	0.0236	0.01247	0.01919	0.02345	0.01981	0.01391	0.01519	0.01897	0.02052	0.01973	0.01095	0.01927	0.0168	0.01921	0.01998	0.01785	0.01565	0.02248	0.02031	0.02215	0.0215	0.01887	0.0233	0.02292	0.02188	0.01935	0.01716	0.02275	0.02163	0.02255	0.01674	0.01852	0.02047	0.02243	0.02327	
3680	0.00827	0.0181	0.02033	0.01726	0.01799	0.02027	0.01361	0.01473	0.01888	0.01879	0.02111	0.01198	0.01752	0.02213	0.01946	0.01417	0.01387	0.01818	0.01989	0.01915	0.0112	0.01888	0.01606	0.01888	0.01901	0.01603	0.01478	0.02142	0.01941	0.02142	0.02071	0.01942	0.02243	0.02241	0.02128	0.017	0.01621	0.02199	0.02105	0.02173	0.01652	0.01789	0.0196	0.0225	0.02272	
3720	0.00918	0.01712	0.0196	0.01665	0.01762	0.0194	0.01239	0.01421	0.01812	0.01822	0.01821	0.01153	0.01604	0.0208	0.01892	0.01463	0.01263	0.01751	0.01941	0.01863	0.01125	0.01867	0.01558	0.01853	0.01807	0.0144	0.01381	0.0203	0.01856	0.02065	0.01991	0.01995	0.02142	0.0218	0.02055	0.01454	0.01551	0.02113	0.02054	0.0208	0.0166	0.01754	0.0189	0.02255	0.02206	
3760	0.00993	0.01631	0.01887	0.01606	0.01713	0.0186	0.01158	0.01382	0.01722	0.01764	0.01528	0.01111	0.01475	0.01954	0.01812	0.01512	0.01156	0.01688	0.01898	0.01807	0.011	0.01854	0.01527	0.01801	0.01708	0.01308	0.01282	0.01907	0.01766	0.01979	0.01918	0.02029	0.02015	0.02095	0.01959	0.01225	0.01503	0.02004	0.01985	0.0196	0.01687	0.01735	0.01821	0.02224	0.02101	
3800	0.01037	0.01575	0.01813	0.01541	0.01648	0.01798	0.01118	0.01351	0.01615	0.017	0.01266	0.01073	0.01362	0.01836	0.01703	0.01545	0.01073	0.0162	0.0185	0.01738	0.01044	0.01839	0.01502	0.01722	0.01598	0.01212	0.0119	0.01775	0.01662	0.01876	0.01856	0.02033	0.01856	0.01972	0.01829	0.01037	0.01472	0.01863	0.01869	0.01798	0.01721	0.0172	0.01737	0.02127	0.01933	
3840	0.01042	0.01546	0.01737	0.01468	0.01567	0.0176	0.01115	0.01322	0.01494	0.01627	0.01064	0.01038	0.01261	0.01723	0.01569	0.0155	0.01021	0.01542	0.0179	0.01653	0.00966	0.01815	0.01471	0.01611	0.01473	0.01154	0.01113	0.01638	0.0154	0.01754	0.01803	0.02001	0.01669	0.0181	0.01661	0.00907	0.01449	0.01685	0.01686	0.01587	0.01752	0.01698	0.01625	0.01944	0.01688	
3880	0.01014	0.01534	0.01661	0.01386	0.01477	0.01745	0.01141	0.01294	0.01367	0.01547	0.00939	0.01005	0.01171	0.01615	0.0142	0.01522	0.00999	0.01455	0.01714	0.01553	0.00878	0.01777	0.01428	0.01474	0.01335	0.01129	0.01055	0.01503	0.01397	0.01613	0.01758	0.0194	0.01467	0.01612	0.01461	0.00838	0.01425	0.01476	0.01433	0.01329	0.01778	0.01661	0.01477	0.01672	0.01367	
3920	0.00961	0.01526	0.01589	0.01301	0.01389	0.01747	0.01185	0.01266	0.01245	0.01467	0.00892	0.00975	0.0109	0.0151	0.0127	0.01465	0.01003	0.01361	0.01625	0.01445	0.00793	0.01726	0.01373	0.01321	0.01191	0.01129	0.01013	0.01379	0.01242	0.0146	0.0172	0.0186	0.01269	0.01394	0.01241	0.00827	0.01392	0.01253	0.01122	0.01039	0.01796	0.01607	0.01298	0.0133	0.00992	
3960	0.00899	0.01508	0.01526	0.01221	0.01317	0.0176	0.01236	0.01238	0.01143	0.01396	0.00915	0.00948	0.01023	0.01413	0.01137	0.0139	0.01026	0.01272	0.01532	0.0134	0.00722	0.01667	0.01312	0.01174	0.01055	0.01142	0.00982	0.01279	0.01087	0.01307	0.0169	0.01777	0.01096	0.01181	0.01021	0.00857	0.01345	0.01039	0.00785	0.00745	0.01811	0.01542	0.01105	0.00958	0.00604	
4000	0.00841	0.01475	0.01477	0.01158	0.01274	0.01773	0.01282	0.01214	0.01077	0.01345	0.00985	0.00926	0.00976	0.01334	0.01041	0.01312	0.01056	0.01199	0.01446	0.01253	0.00674	0.01608	0.01256	0.01055	0.00943	0.01161	0.00955	0.01212	0.00952	0.01173	0.0167	0.01707	0.00972	0.01003	0.00828	0.00911	0.01286	0.00865	0.00471	0.00485	0.01824	0.01476	0.00926	0.00612	0.00261	
